# Dissolving Silver Nanoparticles Modulate the Endothelial Monocyte-Activating Polypeptide II (EMAP II) by Partially Unfolding the Protein Leading to tRNA Binding Enhancement

**DOI:** 10.3390/ijms27020605

**Published:** 2026-01-07

**Authors:** Lesia Kolomiiets, Paulina Szczerba, Wojciech Bal, Igor Zhukov

**Affiliations:** Institute of Biochemistry and Biophysics, Polish Academy of Sciences, 02-106 Warsaw, Poland; pszczerba@ibb.waw.pl (P.S.); wbal@ibb.waw.pl (W.B.)

**Keywords:** aminoacyl synthetase, tRNA, EMAP II, silver nanoparticles, UV-Vis and fluorescence spectroscopy, heteronuclear NMR spectroscopy

## Abstract

Metal nanoparticles (NP) are increasingly used in biomedical applications. Among them, silver NPs (AgNPs) are used as active components in antibacterial coatings for wound dressings, medical devices, implants, cosmetics, textiles, and food packaging. On the other hand, AgNPs can be toxic to humans, depending on the dose and route of exposure, as agents delivering silver to cells. The cysteine residues are the primary molecular targets in such exposures, due to the high affinity of Ag^+^ ions to thiol groups. The Endothelial monocyte-activating polypeptide II (EMAP II), a cleaved C-terminal peptide of the intracellular aminoacyl-tRNA synthetase multifunctional protein AIMP1, contains five cysteines exposed at its surface. This prompted the question of whether they can be targeted by Ag^+^ ions present at the AgNPs surface or released from AgNPs in the course of oxidative metabolism of the cell. We explored the interactions between recombinant EMAP II, tRNA, and AgNPs using UV-Vis and fluorescence spectroscopy, providing insight into the effects of AgNPs dissolution kinetics on interaction EMAP II with tRNA. In addition, the EMAP II fragments binding to intact AgNPs were established by heteronuclear ^1^H-^15^N HSQC spectra utilizing a paramagnetic probe. Structural analysis of the EMAP II reveal that the 3D structure of protein was destabilized (partially denatured) by the binding of Ag^+^ ions released from AgNPs at the most exposed cysteines. Surprisingly, this effect enhanced tRNA affinity to EMAP II, lowering its $K_{d}$. The course of the EMAP II/tRNA/AgNP reaction was also modulated by other factors, such as the presence of Mg^2+^ ions and TCEP, a thiol-group protector used to mimic the reducing conditions of the cell.

## 1. Introduction

Aminoacyl-tRNA synthetases (ARSs) are an important class of enzymes participating in protein synthesis by activating amino acids and linking them to their cognate transfer RNAs (tRNAs). In mammals, ARSs exist in two forms, as free polypeptides and as elements of the multi-tRNA synthetase complex (MSC). MSC consists of nine ARSs and three ARS-interacting multifunctional proteins (AIMP1/p43, AIMP2/p38, and AIMP3/p18) [1,2,3]. AIMPs are non-enzymatic factors, providing a scaffolding during the MSC assembly and maintaining its structural stability [4,5]. The formation of the ARS complex at the core of MSC is considered a canonical function of AIMP1/p43 [1,6]. This protein, and AIMPs in general, also have non-canonical functions, participating in apoptosis, inflammatory processes, DNA repair, immune regulation, nervous system functions, viral replication, genome stability, and anti-angiogenic properties [7,8,9,10]. Under a range of conditions, including cellular stress, AIMP1/p43 is released from the ARS complex and secreted as a proinflammatory cytokine [11,12].

The C-terminal region of AIMP1/p43 is a precursor of endothelial monocyte-activating polypeptide-II (EMAP II) [4,13,14]. This polypeptide is responsible for tRNA binding, and is pro-inflammatory and anti-angiogenic [15]. EMAP II induces pro-coagulant activity on the surface of endothelial cells, increases expression of E-and P-selectins and tumor necrotic factor receptor-1, directs migration of monocytes and neutrophils and induces apoptosis in cultured endothelial cells [14,15,16]. The amino acid sequence of EMAP II, comprised residues 146–312 of UniProt Q12904 entry, is provided in Supplementary Information (Figure S1).

Nanoparticles are colloidal metal particles with a diameter of 1 to 100 nm. Among them, silver nanoparticles (AgNPs) are increasingly used in biomedicine, with applications related to molecular imaging, drug delivery, diagnostics, but foremost the production of medical devices and materials with antimicrobial properties [17,18,19]. On the other hand, AgNPs can be toxic to mammalian cells and organs in a size-dependent manner, with the smallest ones doing most harm [20]. This is due to their ability to penetrate the cellular membrane by passive transport. The exposure to AgNPs results in the impairment of mitochondrial function, DNA damage, and apoptosis [21,22]. When injected into the bloodstream, AgNPs cause disruption of the blood–brain barrier and damage the neurons in vivo [23]. This can also occur with other exposure routes, making AgNPs a potential systemic toxin [24].

Following the cell entrance, AgNPs undergo gradual dissolution into Ag^+^ ions, which preferably bind to thiol groups [25]. Molecular and cellular studies identified the formation of Ag-Cys clusters in zinc finger proteins [26,27,28,29] and metallothioneins [30,31]. However, in principle, all proteins bearing surface Cys residues can serve as Ag^+^ targets.

EMAP II contains five cysteine residues exposed at its surface [32], and we assumed that it could interact with Ag^+^ ions, both free and present at the surface of dissolving AgNPs (Figure S2). In the present study we explored the interactions between recombinant EMAP II, tRNA and AgNPs using UV-Vis and fluorescence spectroscopies. The structural aspects of these interactions were followed by heteronuclear ^1^H-^15^N HSQC NMR spectra. The obtained data indicate that protein biosynthesis may be impaired upon the cell exposure to AgNPs. TCEP, a thiol-protecting phosphine, was used to mimic the reducing conditions inside the cell. The experiments performed in the absence of TCEP mimicked the oxidative stress conditions.

Our experiments were performed for 165 pM AgNPs, 1–3 μM recombinant EMAP II protein and 1–3 μM tRNA. The AgNPs concentrations was chosen in correspondence with previous experiments. The full dissolution of these AgNPs would yield 40 μM Ag^+^ ions (see Supplementary Materials for details of calculations), which is consistent with studies of cell exposure to similar citrate-coated AgNPs [29]. The 1 μM concentration of tRNA is within the physiological range (taking 1 pL for a small eukaryotic cell one obtains ca. 600,000 tRNA copies per cell), which is consistent with the literature [33]. A broader account of intracellular concentrations and their limitations was described [34].

## 2. Results

### 2.1. Choice of Experimental Conditions

The identity of recombinant EMAP II was confirmed by MS (Figure S1) and NMR (Figure S3). Silver nanoparticles were characterized using DLS (Figure S4). Further details are provided in the Section 4 and the introductory paragraph of Supplementary Materials.

Preliminary experiments were performed in 20 mM HEPES and Tris/HCl buffers. These experiments indicated that EMAP II solutions were significantly less stable in HEPES buffer than Tris (see Figure S5 for melting temperature $T_{1/2}$ determination, Table S1 for the $T_{1/2}$ values and Figure S6 for selected spectra). Therefore, Tris/HCl was chosen for all subsequent studies. Since the aminoacylation process occurs with the participation of ATP, we decided to add ATP to the working buffer [35].

The next component to be optimized was the MgCl_2_ concentration. The Mg^2+^ cations are necessary for the stability of tRNA, but in preliminary experiments we noticed that they alone accelerated the decomposition of AgNPs in a concentration-dependent way (Figure S7). This reaction was monitored at 399 nm—the maximum of absorption for AgNPs. This reactivity can be attributed to the destruction of citrate coating of AgNPs through the formation of the Mg(citrate) complex with $K_{d}$ ∼ 0.5 mM [36]. Accordingly, we found that the concentration of Mg^2+^ ions in the EMAP II-tRNA buffer should be minimized to maintain the stability of AgNPs in respective experiments. In order to optimize it, we titrated the EMAP II/tRNA complex with Mg^2+^ ions using the EMAP II fluorescence signal. The obtained partial signal quenching, presented in Figure S8, occurred along three linear segments, which could be associated to specific, quantitative binding of Mg^2+^ ions to EMAP II/tRNA, followed by the Mg^2+^/ATP binding and the signal stabilization at higher Mg^2+^ concentrations. These experiments allowed us to conclude that the 0.2 mM Mg^2+^ concentration should be both safe for the AgNPs and sufficient to saturate the EMAP II/tRNA complex and the accessory ATP molecules. This concentration was used in all subsequent experiments.

TCEP is a thiol protecting agent routinely used to prevent the formation of adventitious disulfide bridges in proteins. It was thus used as a component of the buffer in samples containing EMAP II. TCEP is, however, an efficient chelator for transition and heavy metal ions [37], and Ag^+^ ions were demonstrated to eagerly form phosphine complexes [38,39]. Figure S9 presents the time dependence of the main absorption band of AgNPs in the optimized buffer with 50 μM TCEP. The AgNPs signal at 399 nm vanished according to the 1st order kinetic law, with $t_{1/2}$ = 37 ± 1 min.

The stability of the EMAP II/tRNA complex in time was followed by tRNA band centered at 257 nm in the UV-Vis spectra (Figure S10). No change of the spectrum was observed over the 10 h incubation time.

The 1 μM EMAP II sample in the working buffer was incubated for 22 h, as demonstrated in Figure S11. A very limited drop of protein fluorescence was observed (7% over 22 h, $t_{1/2}$ = 2 h) without the change of fluorescence maximum. This effect could be due to slight protein adsorption on the cuvette walls. There was no shift of fluorescence maximum, confirming the absence of protein denaturation. The analogous experiment was performed in the presence of equimolar tRNA and indicated an even higher stability of the protein (Figure S12). These two experiments were performed in the absence of TCEP, which further confirmed remarkable stability of EMAP II under the working conditions used in this study. The binding of tRNA did not affect the thermal stability of EMAP II, as evidenced by the unchanged melting temperature of 45 °C (Table S1).

### 2.2. Interactions of AgNPs with EMAP II and tRNA With and Without TCEP

**Figure 1:**
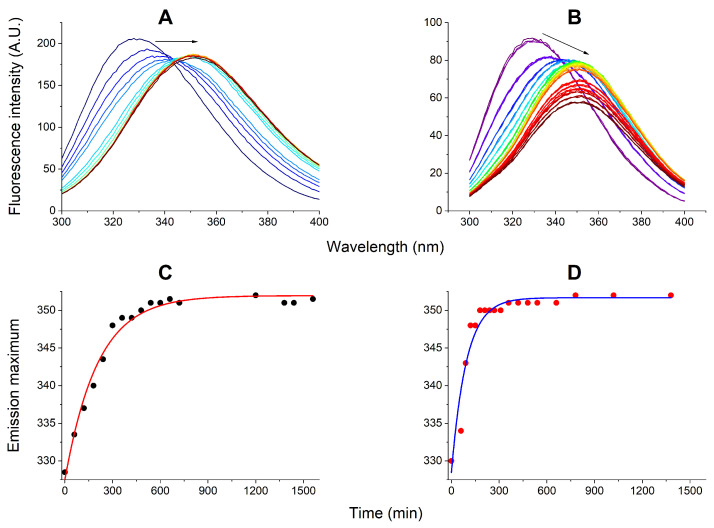
The kinetics of EMAP II interaction with 165 pM AgNPs in the optimized buffer monitored by fluorescence spectroscopy over the period of 26 h. Fluorescence spectra of 1 μM EMAP II were recorded periodically in 20 mM Tris, 0.2 mM MgCl_2_, 5 μM ATP, pH 8.0, and the presence (**A**) or absence (**B**) of 50 μM TCEP. Arrows mark the general direction of change. The kinetic plots of emission maximum wavelengths for these reactions are presented in panels (**C**) and (**D**), respectively. Lines mark the 1st order kinetic fits to these data.

The interactions of AgNPs with EMAP II, tRNA, and their complex were investigated for 1 μM EMAP II dissolved in the optimized buffer described above and 1 μM tRNA, where appropriate. Some protein stability experiments were performed in the presence of 50 μM TCEP, taking into account its detrimental effect on AgNPs. The fluorescence and UV-Vis experiments were performed in parallel with the same samples. Figure 1 presents the effect of incubation with 165 pM AgNPs on the EMAP II stability, monitored using the fluorescence of its Trp residues. The redshift of fluorescence emission indicated the increased exposure of Trp indole rings to solution [40]. Therefore, the presence of AgNPs caused the protein denaturation. In the presence of TCEP (Figure 1A) the spectral changes exhibited an isosbestic point, along the shift of emission maximum from 330 to 352 nm, while the emission intensity only slightly decreased. Hence, in this case the EMAP II the unfolding occurred between two defined conformations, the folded (native) and partially unfolded ones. In the absence of TCEP (Figure 1B), the second phase of EMAP II denaturation was observed. It was characterized by the significant decrease of fluorescence intensity, but the emission maximum remained at 352 nm. Therefore, the fluorescence emission maximum plotted against time of incubation could be used to track the first denaturation phase specifically. These plots are presented in Figure 1C,D for the reactions with and without TCEP, along with 1st order kinetic fits, which yielded $t_{1/2}$ values of 143 ± 10 and 65 ± 9 min, respectively.

The emission wavelength changes in this experiment were identical with those observed during the thermal denaturation of EMAP II (Figure S6), but in the latter the redshift was accompanied with the increase of fluorescence. One can thus assume that TCEP limited but did not fully prevent the EMAP II oxidation. Both protein unfolding and putative oxidation apparently occurred in parallel, but the overall process was about twice faster without TCEP.

**Figure 2:**
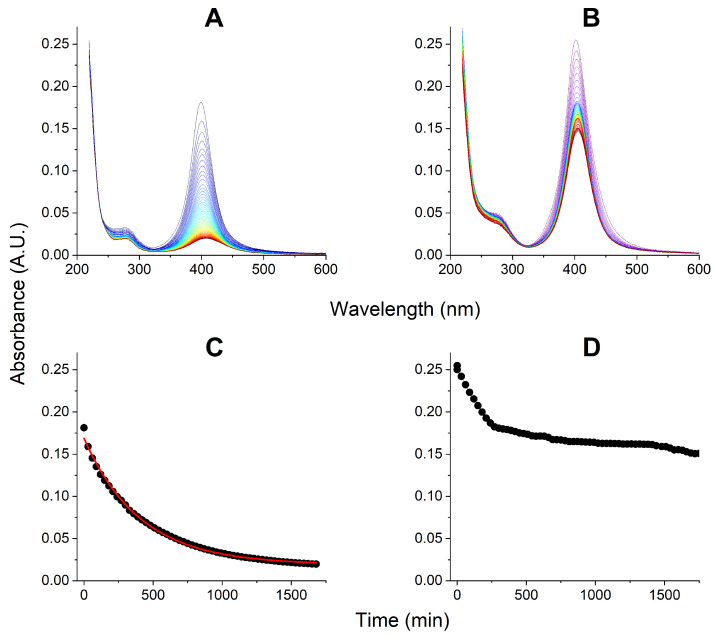
The kinetics of decay of 165 pM AgNPs in the presence of 1 μM EMAP II in 20 mM Tris, 0.2 mM MgCl_2_, 5 μM ATP, pH 8.0, and the presence (**A**) or absence (**B**) of 50 μM TCEP. The kinetic plots at 399 nm for these reactions are presented in panels (**C**) and (**D**), respectively. The 1st order kinetic fit in panel (**C**) is provided as red line.

The parallel experiment followed by UV-Vis spectroscopy demonstrated the fate of AgNPs in these experiments, as presented in Figure 2. In the presence of TCEP, the AgNPs decay followed the 1st order kinetics with and half-time $t_{1/2}$ = 280 ± 5 min. Without TCEP, the slight initial signal decay progressed for ca. 4.5 h, followed by a very slow, nearly linear dissolution, which still left more than 60% of AgNPs intact after 24 h of incubation.

The effect of tRNA on the stability of AgNPs is presented in Figure S13. In the presence of TCEP the AgNPs decayed with the approximate 1st order kinetics, and the half-time $t_{1/2}$ = 44.5 ± 1.5 min, only slightly slower than the absence of tRNA. In the absence of TCEP the decay was bilinear, like for the AgNPs interaction with EMAP II, but with the shorter first phase, ca. 60 min, followed by a longer period of near stability, with over 80% of AgNPs retained after 24 h.

Finding out that the dissolution of AgNPs to Ag^+^ ions occurred during partially or fully during incubations presented above, we also exposed EMAP II to 40 μM Ag^+^ provided as AgNO_3_. The results are presented in Figure S14. EMAP II underwent denaturation analogous to that observed in AgNPs exposures. In the absence of TCEP (Figure S14A) the reaction was biphasic, consisting of the fluorescence redshift, followed by reduction of fluorescence intensity without a further shift of the emission maximum. In its presence (Figure S14B), only the first phase was observed. Its kinetics was quantified my fitting the 1st order rate law to the emission wavelength maximum for both experiments. The obtained $t_{1/2}$ values were 82 ± 3 min with TCEP and 23 ± 2 min without TCEP. In the presence of tRNA, the first phase of the reaction was immediate, followed by a slower second phase, which initially followed the 1st order kinetics with $t_{1/2}$ 30 ± 2 min (Figure S15).

### 2.3. Interactions of the EMAP II-tRNA Complex with AgNPs

**Figure 3:**
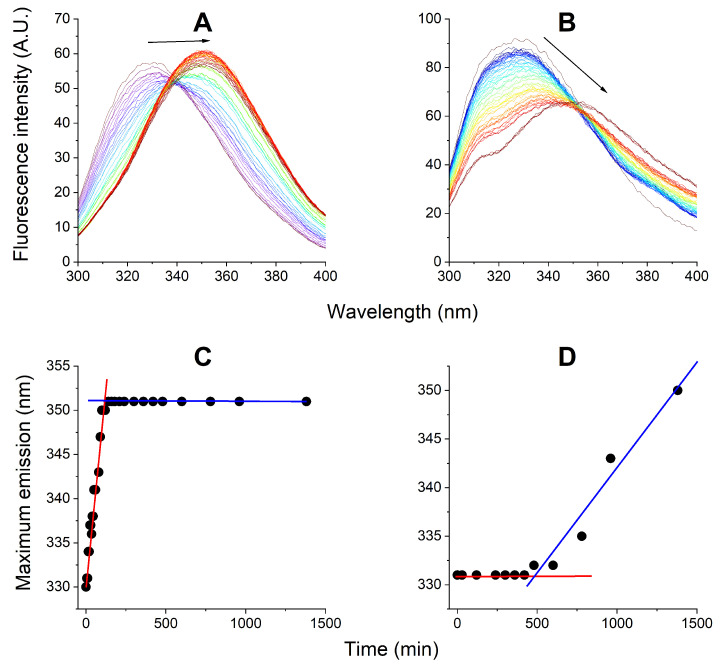
The kinetics of EMAP II/tRNA interaction with 165 pM AgNPs in the optimized buffer monitored by fluorescence spectroscopy over the period of 24 h. Fluorescence spectra of 1 μM EMAP II were recorded periodically in 20 mM Tris, 0.2 mM MgCl_2_, 5 μM ATP, pH 8.0, in the presence (**A**) or absence (**B**) of 50 μM TCEP. Arrows mark the general direction of change. The kinetic plots of emission maximum wavelengths for these reactions are presented in panels (**C**) and (**D**), respectively. Lines mark the linear approximations in sections of kinetic plots.

Next, the analogous protocols were used to follow the interactions of 165 pM AgNPs with the EMAP II/tRNA complex, also with and without TCEP. Like above, the fate of the protein was followed by fluorescence spectroscopy (Figure 3). In the presence of TCEP the apparently monophasic process was observed, consisting of the emission redshift down to 352 nm, without the loss of signal. It was qualitatively similar to that observed in the absence of tRNA, but nearly five-fold faster. In its absence, the Trp fluorescence emission remained nearly intact for ca. 7 h, followed by a gradual decrease and redshift.

**Figure 4:**
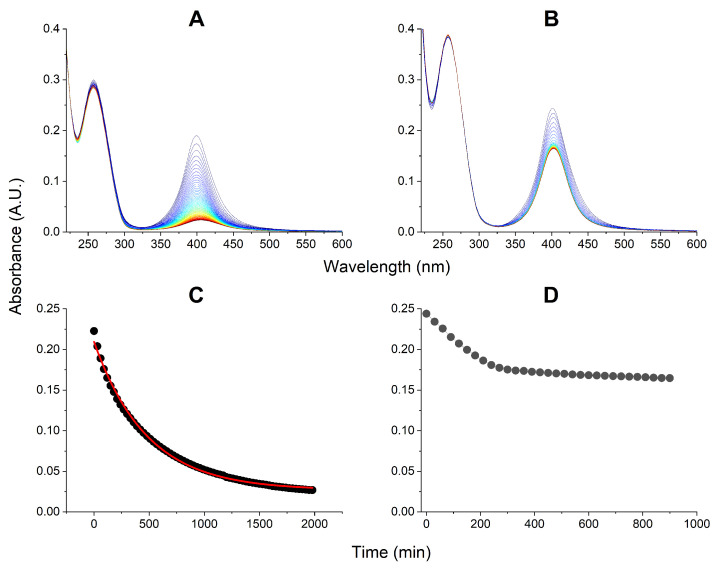
The kinetics of decay of 165 pM AgNPs in the presence of 1 μM EMAP II and 1 μM tRNA in 20 mM Tris, 0.2 mM MgCl_2_, 5 μM ATP, pH 8.0, and the presence (**A**) or absence (**B**) of 50 μM TCEP. The kinetic plots at 399 nm for these reactions are presented in panels (**C**) and (**D**), respectively. The 1st order kinetic fit in panel (**C**) is provided as red line.

The evolution of AgNPs, followed in parallel, is presented in Figure 4. In the presence of TCEP a uniform 1st order kinetic process was observed, resulting in a practically complete decay of AgNPs. A similar process was seen in the absence of tRNA (Figure 2), but the presence of tRNA decelerated it (increased $t_{1/2}$ from 280 ± 5 min to 327 ± 7 min).

In the absence of TCEP, there was much less decay of AgNPs and the process was very similar to that without tRNA. After the initial linear phase of AgNPs decomposition that took ca. 4 h, very slow further decay followed which left the 60% of initial AgNPs amount after 15 h.

### 2.4. Determination of the tRNA Binding Affinity to Native and Denatured EMAP II

**Figure 5:**
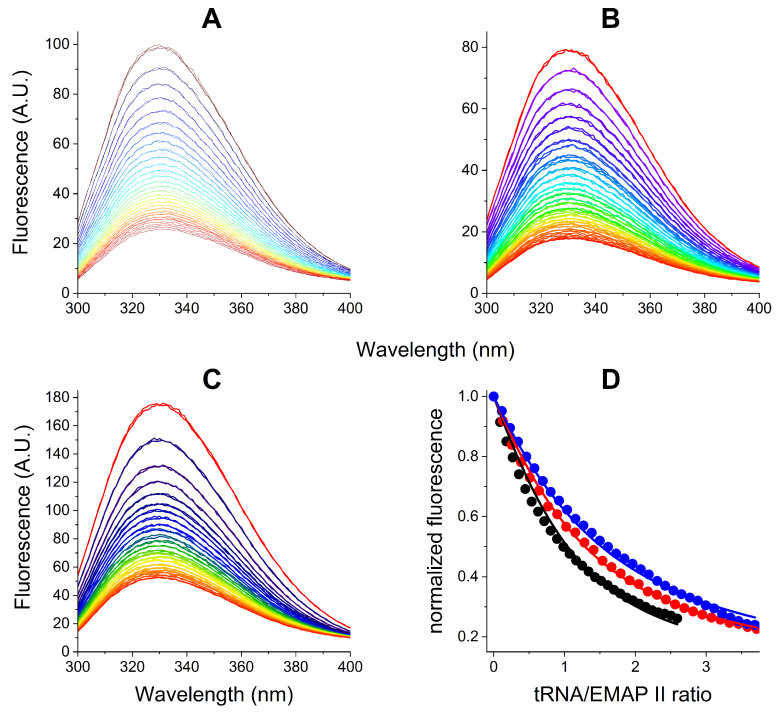
Fluorescence titrations of EMAP II at concentrations of 5 μM (**A**), 3.5 μM (**B**) and 2.5 μM (**C**) with tRNA in the presence of 20 mM Tris/HCl, 0.2 mM MgCl_2_, 5 μM ATP, and 50 μM TCEP, pH 8. The spectra were recorded in triplicate in 5 min intervals for the starting sample and after each tRNA addition. No kinetic phenomena were observed. The affinity constant was calculated by the global fit of the binding isotherm to all three experiments (**D**). Black, red, and blue symbols correspond to normalized fluorescence at 330 nm of 5 μM, 3.5 μM and 2.5 μM EMAP II, respectively.

To confirm and quantify the binding between EMAP II and tRNA, we conducted a series of EMAP II titrations with tRNA, monitored by changes of Trp fluorescence. The concentrations of EMAP II and tRNA in these titrations were in the range of 2.5 to 5 μM, and 0 and 13 μM, respectively. Both EMAP II and tRNA were dissolved in the optimized buffer (20 mM Tris/HCl, 0.2 mM MgCl_2_, 5 μM ATP, pH 8) with added 50 μM TCEP, to assure the protein integrity. The fluorescence spectra and global fit of three independent titrations is presented in Figure 5. The addition of tRNA resulted in the partial quenching of EMAP II tryptophan fluorescence. A single binding site model with 1:1 molar stoichiometry provided the best correspondence to the experimental data. The fitted $K_{a}=4.1\pm 0.2\cdot 10^{5}\;M^{-1}$ was obtained (corresponding to $LogK_{a}=5.62\pm 0.03$, and $K_{d}=2.4\pm 0.2$ μM).

**Figure 6:**
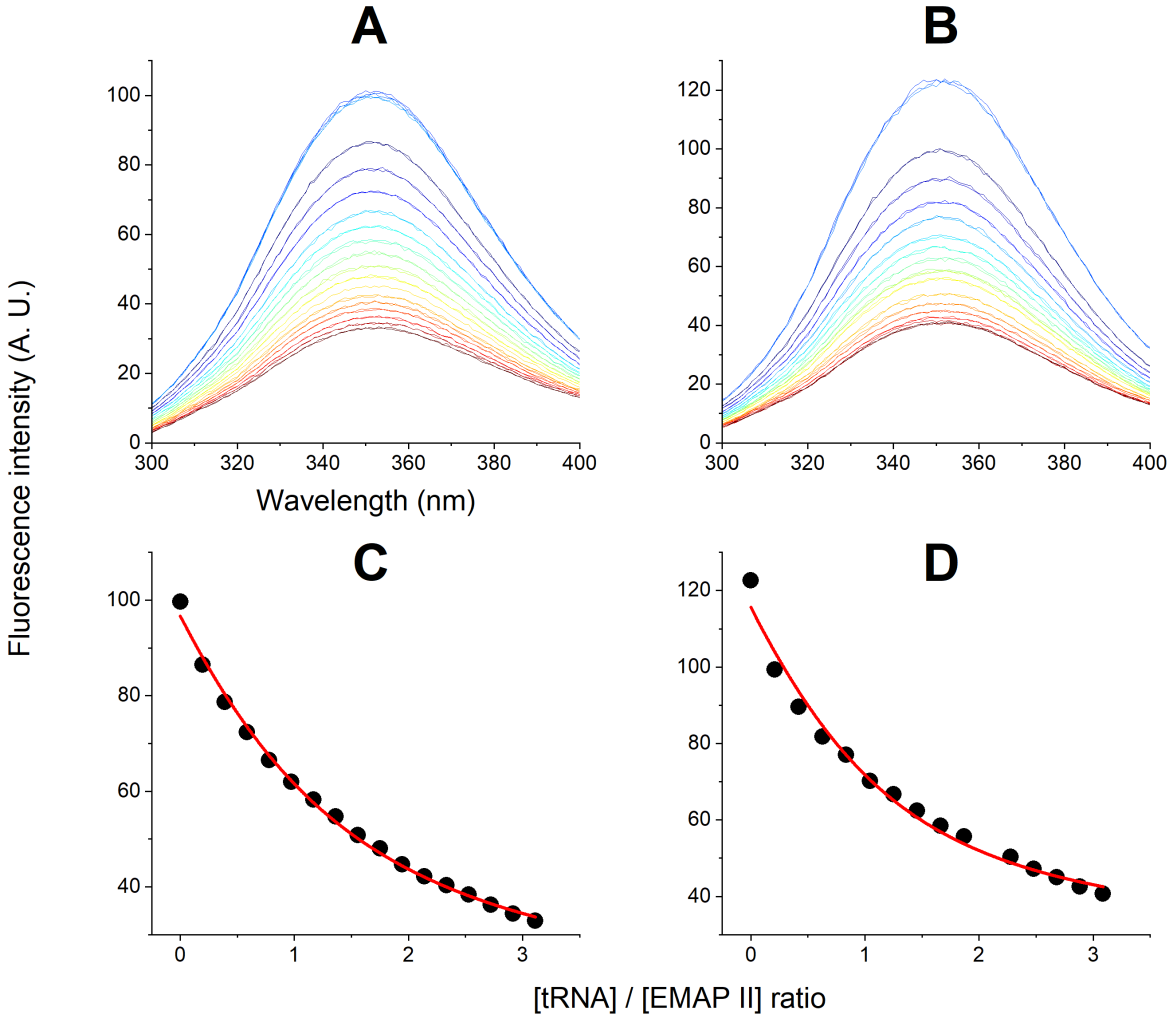
Examples of tRNA titrations of (**A**) 1.63 μM EMAP II denatured by 165 pM AgNPs in the optimized buffer in the presence of 50 μM TCEP and (**B**) 1.85 μM EMAP II in the absence of TCEP; (**C**,**D**): respective titration curves derived from fluorescence maxima (symbols) and the fitting curves (lines).

Next, we investigated the effect of AgNPs on tRNA binding to EMAP II. In these experiments EMAP II was preincubated with AgNPs for at least 24 h, to assure full saturation of all AgNP-related interactions. The binding process was followed again by the quenching of the fluorescence signal of EMAP II. The preincubations and titrations were performed in the optimized buffer with and without 50 μM TCEP. Nine separate experiments were done, three with TCEP and six without. Representative examples are presented in Figure 6. Strikingly, the denaturation of EMAP II increased its affinity for tRNA ca. 3-fold. The average log $K_{a}$ value from all nine titrations is 6.1 ± 0.1, corresponding to $K_{d}=0.8\pm 0.3$ μM vs. 2.4±0.2 μM determined for the native protein. No effect of TCEP on this interaction could be discerned (see Table S2 for all titration data and Figure S16 for the whole titration dataset). In a control experiment, a 1 μM sample of EMAP II was incubated for 24 h in the working buffer containing 40 μM AgNO_3_ instead of AgNPs, followed by tRNA titration. The resulting spectra and titration curve are presented in Figure S17. The determined $K_{d}$ = 1.1 ± 0.2 μM, which is within the statistical spread of values obtained for AgNPs.

### 2.5. Structural Information on EMAP II Interaction with AgNPs Provided by NMR Spectroscopy

**Figure 7:**
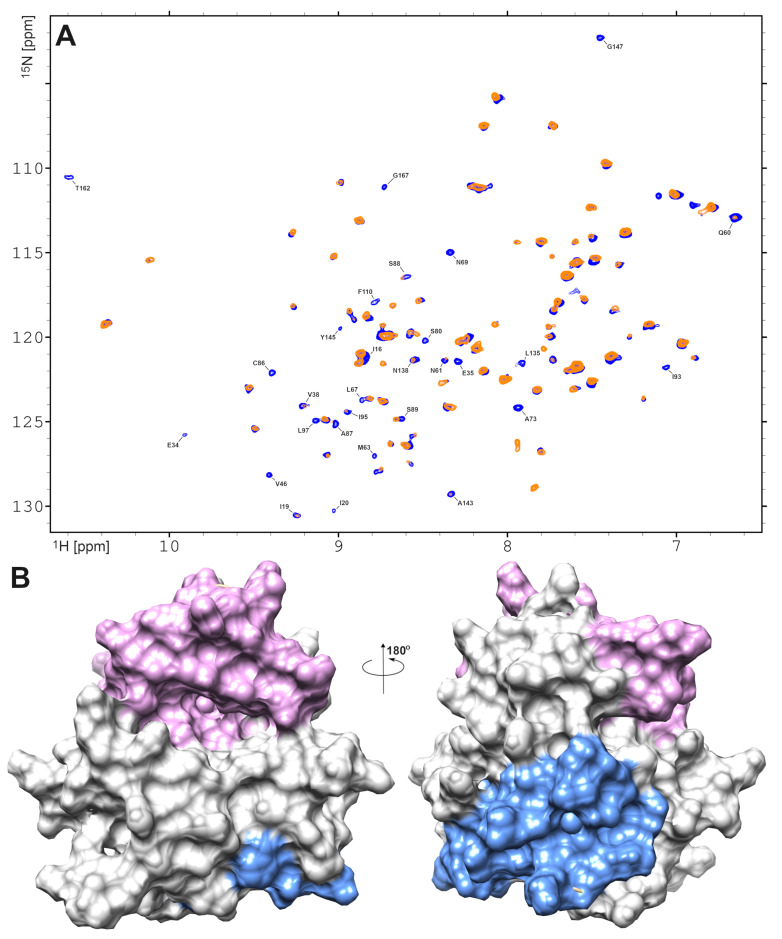
(**A**) The ^1^H-^15^N HSQC spectrum of EMAP II recorded in the presence of AgNPs, and a small amount of paramagnetic species, CrAcAc. The blue and yellow-coded signals mark the spectra recorded in the absence and presence of the paramagnetic relaxation agent. (**B**) Two regions on EMAP II surface which are, more probably, interacted with AgNPs as follows from paramagnetic NMR data.

Information about residues that interacted with AgNPs was extracted from a ^1^H-^15^N HSQC experiment, where a small amount (10 μM) of paramagnetic species, CrAcAc, was added to the freshly prepared sample containing EMAP II and AgNPs. This resulted in dramatic increase of the linewidth of amide group protons in residues exposed to solvent, due to paramagnetic relaxation. This resulted in the disappearance of cross-peaks belonging to these residues from the ^1^H-^15^N HSQC spectrum (Figure 7A).

A small amount of paramagnetic CrAcAc substantially decreases the number of signals in the ^1^H-^15^N HSQC spectrum (Figure 7A). The remaining signal reveals the amide groups, which are buried in the hydrophobic core and have no access to solvent. The inspection of the presented data revealed two corresponding structural fragments that characterize possible binding of AgNPs and/or oligomerization of EMAP II. The first comprised the interface formed by three loops: Lys24–Val33, Val38–Leu56, and Lys71–Met85. The second motif includes Leu96–Thr109, His136–Ala143, and Val148–Ala160 fragments, which are located on the other side of the EMAP II 3D structure. Interestingly, the His136–Ala143 loop neighbors the tRNA binding motif, providing the structural basis for discussing the interference of EMAP II tRNA binding and EMAP II interaction with AgNPs (Figure 7B) shows the proposed positioning of EMAP II and the AgNPs.

## 3. Discussion

### 3.1. Initial EMAP II Interactions with AgNPs

The DLS and zeta potential measurements indicated that the AgNPs underwent immediate coating upon the addition of EMAP II. Taking into account the increased nanoparticle diameter, from 33.9 to 48.35 nm due to this process, and the size of EMAP II molecule one can estimate the average number of EMAP II molecules in the AgNP corona. The approximate diameter of EMAP II estimated from its three-dimensional structure provided by NMR is 6 nm (pdb 8ONG) [32]. Therefore, one can assume that EMAP II formed a single layer around a citrate-coated AgNP. We assume such initial structure including citrate ions, because the AgNPs dissolution at low Mg^2+^ concentration, where the citrate coat remained intect, is a time-consuming process (Figure S7F), and the corona formed within a few minutes of DLS sample preparation. Using a spherical shape approximation for both the AgNPs and one layer of EMAP II, we calculated that such coating would include ca. 188 protein molecules. With the formal AgNPs molar concentration of 165 pM, this results in ca. 31 nM EMAP II in the corona. Therefore, initially ca. 3% of the EMAP II total concentration were present in the AgNP corona in the experiments monitored by UV-Vis and fluorescence spectroscopies, and this fraction decreased along the AgNPs decay.

### 3.2. Kinetic Experiments: EMAP II Interactions with AgNPs and AgNP Decay

The dissolution of AgNPs is based on Ag^0^ oxidation to Ag^+^ by ambient oxygen. This process is prevented/slowed down mechanically by the citrate coating, as evidenced by the Mg^2+^ experiments presented in Figure S7. The changes in EMAP II caused by AgNPs monitored in kinetic experiments must therefore belong to two mechanisms: direct interactions with AgNPs, going through a bottleneck of limited AgNP corona occupancy and indirect interactions with Ag^+^ ions mobilized from the AgNPs.

On the other hand, the pattern of AgNPs dissolution depends on the presence of EMAP II. In general, the main pathway of this process, involves the diminishing of the 399 nm signal, without its significant shift (Figure 2, Figure 4, Figures S7 and S13). This means that AgNPs with metal core sizes different from the original 20 nm never accumulate during the AgNPs dissolution. We should see the AgNPs band blueshifting for smaller AgNPs and redshifting for larger/aggregated AgNPs. As this did not happen, we can state that once the protective citrate coat of a given AgNP became compromised, that AgNPs dissolved fast into Ag^+^ ions.

Two kinetic modes of AgNPs dissolution were observed in the presence of EMAP II, depending on the presence of TCEP in the studied solutions. In its absence, a bilinear slow kinetics was observed (Figure 2D), while in its presence the complete AgNPs decay followed a pseudo-first order regime, with the reaction half-time of 280 min. Qualitatively the same patterns were observed also in the presence of tRNA alone (Figure S13), and co-presence of EMAP II and tRNA (Figure 4). This allows us to conclude that TCEP, when present, controlled the fate of AgNPs. TCEP is a phosphine widely used to maintain protein thiols in the reduced state, but is also a strong chelator of transition and heavy metal ions. The stability of Ag^+^/TCEP complexes has not been studied directly, but it is high, which can be inferred from the NMR spectroscopic parameters of other Ag^+^-phosphine complexes [38,39]. The TCEP concentration in our experiments was higher than the total Ag concentration (50 μM vs. 40 μM). Therefore, despite its reducing properties, TCEP enhanced the AgNPs oxidation, by sequestering the resulting Ag^+^ ions without reducing them back to metal. The pseudo-first order character of this process indicates the active participation of TCEP in the reaction, likely by direct TCEP binding to AgNPs. Such process is obviously enhanced by the fact that TCEP is a tricarboxylic molecule, like citrate, and can easily replace it.

### 3.3. Kinetic Experiments: EMAP II Denaturation Mechanisms and AgNPs

EMAP II underwent partial, two component denaturation in the presence of AgNPs. Other buffer components did not elicit such effect. This process was monitored by fluorescence spectroscopy. Depending on the experimental conditions and the phase of reaction either the redshift of EMAP II Trp fluorescence was observed without a significant signal decrease, or the fluorescence quenching was observed without a signal maximum movement. By a comparison with the thermal denaturation experiment (Figures S5 and S6), the redshift can be assigned to the partial opening of the EMAP II structure, exposing the hydrophobic pocket where Trp residue to the solvent. It was previously reported that the binding of tRNA to EMAP II occurs with the participation of amino acid residues located near Trp [40]. The positively charged tRNA-binding motif of the α-helix of EMAP II ^122^NPKKKEW^128^ corresponds to the negative charge of the tRNA. It is likely that unfolding of the EMAP II molecule leads to better binding of the indicated amino acid residues to tRNA, as the accessibility of the tRNA-binding motif increases.

The fluorescence quenching in the open EMAP II structure could be due to the Ag^+^ binding to the SH groups of Cys residues. Trp or Tyr fluorescence alteration (quenching or enhancing) due to the formation of heavy metals at nearby thiol groups is a known phenomenon [41,42]. However, the alternative quenching mechanism due to intraprotein disulfide formation cannot be excluded.

These two processes overlap to some extent, but we were able to separate the first of them by plotting the fluorescence maximum against the incubation time. As presented in Figure 1B,D, in samples containing just EMAP II and AgNPs the protein opening phase was completed within 6 h, and was followed by the quenching phase. Judging from the AgNPs dissolution kinetics presented in Figure 2B,D, the accumulation of Ag^+^ ions reached about 10 μM, more than enough to saturate the EMAP II thiols. In the presence of TCEP, the EMAP II opening phase was slower, completed after about 10 h, and the quenching phase was not detected during the incubation extended to over 24 h. The parallel AgNPs dissolution process was continuous, and reach near complete dissolution at 24 h. These facts can be interpreted by assuming that the EMAP II opening phase was due to the protein interactions with the AgNPs, and the quenching phase was prevented by sequestered most of Ag^+^ ions by TCEP.

The key supporting experiment helping with the interpretation of these data is provided in Figure S14. In this experiment the EMAP II sample was exposed to 40 μM Ag^+^ ions, equivalent to the total dissolution of AgNPs. In the presence of TCEP there was very little fluorescence quenching, and the spectral redshift followed the pseudo-1st order rate law, like for AgNPs, but was ca. 2 times faster. In the absence of TCEP the process was biphasic, but its opening phase took 3 times less time than with AgNPs. Therefore, we can safely conclude that despite the efficient coating of AgNPs by EMAP II, the observed denaturation effects were solely due to the action of Ag^+^ ions. We can thus assign the opening phase, akin to thermal denaturation, to the Ag^+^ binding to the protein, moderated kinetically by competition from TCEP. Without TCEP, this process is faster, but the absence of TCEP induces the second phase of denaturation which is possibly due to oxidation of thiol groups, despite the presence of strongly thiol binding Ag^+^ ions. Some oxidation might be attributed AgNPs which were demonstrated as a source of reactive oxygen species [43]. The complexation of EMAP II by tRNA apparently exposed the thiol groups to solution. This resulted in the immediate binding of Ag^+^ ions, followed by further denaturation as in the absence of tRNA (Figure S15).

### 3.4. Kinetic Experiments: EMAP II Denaturation Mechanisms and tRNA

The addition of tRNA to the system changed the kinetics of EMAP II and AgNPs degradation quantitatively rather than qualitatively. As without tRNA, the EMAP II denaturation with TCEP was limited to the opening phase, while in its absence the quenching phase followed. However, the kinetic patterns of these processes were changed. Without TCEP, tRNA protected the EMAP II integrity for ca. 7 h, followed by both emission redshift and quenching, occurring simultaneously. This process can be rationalized by the fact that tRNA forms a molecular complex with EMAP II, which apparently slows down the assault of Ag^+^ ions. When we look at the fate of AgNPs in these reactions in Figure 4, we can see that tRNA affected their stability only mildly, compared to the data in Figure 2. This is consistent with the data presented in Figure S11. With TCEP the pseudo-1st order kinetics was replaced by a 0th order (linear) pattern, and the process was completed fast, within less than 2.5 h. This suggests that TCEP may actually serve as Ag^+^ shuttle into the EMAP II/tRNA complex.

### 3.5. The Effect of Ag^+^ on tRNA Affinity for EMAP II

The tRNA titrations of native and silver-denatured EMAP II, presented in Figure 5, Figure 6 and Figure S16, demonstrated strikingly, that the affinity of tRNA to denatured EMAP II is 3-fold higher than to the native protein. This finding explains why after the initial lag period EMAP II underwent both phases of denaturation in the presence of tRNA—this process was guided by the affinity gradient. The lack of effect of TCEP on the titrations indicates that the EMAP II opening process is sufficient to support this interaction.

This finding may have a large impact on the better understanding of oxidative stress and silver toxicity towards the protein synthesis apparatus. This higher affinity resulted in the enhancement of EMAP II denaturation by tRNA, indicating a novel phenomenon where RNA molecules may have deleterious effects by stabilizing non-native protein conformations.

The rates of the described interactions of AgNPs with EMAP II depend strongly on the compositions of experimental solutions, but all occur within 24 h, which is a typical time of intracellular AgNPs dissolution [29]. Therefore, our findings gain additional biological relevance.

## 4. Materials and Methods

Silver nanoparticless with a diameter of 20 nm were obtained from Sigma Aldrich (St. Louis, MO, USA) as 0.02 mg/mL dispersion in aqueous citrate buffer. Ambion™ Yeast tRNA mixture purified from brewer’s yeast was used for tRNA experiments, as provided by the supplier. ATP, DTNB, lysozyme, CaCl_2_ kanamycin, HEPES, Imidazole were obtained from Sigma (St. Louis, MO, USA), D-Glucose, MgCl_2_, MgSO_4_, IPTG, Tris, DTT and NaCl were from Roth (Karlsruhe, Germany), Ni-NTA resin was purchased from Invitrogen (Waltham, MA, USA), and ^15^NH_4_Cl (99% isotopic purity) from Cambridge Isotope Laboratories (Cambridge, UK). TEV protease was previously expressed and purified in our laboratory, using a GenScript construct (Piscataway, NJ, USA).

### 4.1. EMAP II Protein Expression and Purification

The construct pET28a(TEV)-EMAPII (GenScript) was used to transform the *E. coli* BL21(DE3)pLysE strain by heat shock. Transformants were selected in LB agar after incubation for 18 h at 37 °C in the presence of kanamycin (40 μg/mL). A total of 10 mL of overnight culture was sown in 1 L of LB medium containing 40 mg/L kanamycin. The bacterial cells culture was incubated at 37 °C to OD_600_ 0.7–0.9. Protein expression was induced with added isopropyl β-D-1-thiogalactopyranoside (IPTG) to the final concentration of 1 mM. After 3–4 h of induction, the cells were pelleted and lysed, and the EMAP II protein was purified under native conditions. All procedures were performed at 4 °C. Briefly, pelleted cells were lysed with 50 mM Tris buffer, pH 8.0, containing 200 mM NaCl, 10 mM imidazole, and 1 mM DTT in the presence of 1 mg/mL of lysozyme. After sonication, cellular debris was removed by centrifugation before loading on the Ni-NTA slurry (2 mL). Ni-NTA agarose were equilibrated with 50 mM Tris buffer containing 200 mM NaCl, and 1 mM DTT, at pH 8. Protein elution was performed with 50 mM Tris buffer containing 150 mM NaCl, 250 mM imidazole, and 1 mM DTT. After dialysis, the His-Tag was cleaved using TEV-protease for 18 h at 4 °C. Additional protein purification was performed in Superdex 75 10/300 column (GE Healthcare, Hatfield, UK) using size-exclusion chromatography (ÄKTA Pure protein purification system). The A280 value was extrapolated from the protein spectra scanned from 350 to 220 nm on a Varian Cary 50 Bio spectrometer (Varian, Inc., Palo Alto, CA, USA). The specific extinction coefficient 8480 M^−1^ cm^−1^, and relative molecular mass 18,543.55 Da used in the calculations were obtained from the protein amino acid sequence, according to the publicly available method (https://www.expasy.org/, accessed on 29 December 2025). The ESI-MS spectrum (Q-TOF Premier, Waters, Milford, MA, USA) yielded the exact mass of EMAP II protein (18,544 Da) (Figure S1).

### 4.2. ^15^N-Labeled EMAP II Variant

To express the uniformly ^15^N-labeled EMAP II variant for NMR studies 10 mL of Luria broth (LB) starter *E. coli* culture was inoculated in 1 L of LB with kanamycin and the cells were grown at 37 °C until reaching the OD_600_ of ∼1. After that, the cells were precipitated by centrifugation at room temperature at 5000× *g* for 5 min. The cells were then resuspended in freshly prepared optimized minimal medium M9 containing 1 g/L ^15^NH_4_Cl and allowed to grow in it at 37 °C for 1 h. The next step was to induce the expression of the target protein by adding IPTG to the medium, followed by the 4 h incubation at 37 °C. All other steps for preparation of cell lysate and purification were performed as described above. The presence of isotopic labels in the protein was confirmed by mass spectroscopy. EMAP II contains 228 nitrogen atoms, giving a corresponding molecular mass of 18,763 Da.

### 4.3. Dynamic Light Scattering and Zeta Potential

Measurements were performed with a Zetasizer Nano-ZS instrument (Malvern, PA, USA). DLS signals were recorded using the 173° backscatter mode after temperature equilibration for 2 min at 25 °C. Buffer solution (20 mM Tris, 150 mM NaCl, 1 mM DTT) was measured as a control.

### 4.4. Ultraviolet-Visible Spectroscopy

UV-Vis experiments were performed using a Varian Cary 50 Bio spectrophotometer (Varian, Inc., Palo Alto, CA, USA). The spectra were recorded at 25 °C using micro quartz cuvettes with a 1 cm pathlength, with constant stirring. The applied wavelength range was 220–650 nm with the scan rate of 300 nm/min. The AgNPs samples were prepared by diluting 150 μL of the stock solution to a total sample volume of 700 μL. This translates into 40 μM of total silver (AgNPs and released Ag^+^ ions) in the sample. Three or more replicates were performed for kinetic experiments between EMAP II with AgNPs. The spectra were recorded under constant stirring every 30 min, unless stated otherwise.

For determination of the effect of TCEP on the EMAP II interaction with AgNPs, a buffer containing 20 mM Tris, 0.2 mM MgCl2, 5 μM ATP, and 50 μM TCEP was used. For a comparison, the same buffer without TCEP was used. EMAP II concentrations in the micromolar range were determined spectrophotometrically using the specific extinction coefficient 8480 M^−1^cm^−1^ at 280 nm. This extinction coefficient, and protein molecular mass 18,543.5 Da used in the calculations were obtained from the protein amino acid sequence, according to the publicly available method (https://www.expasy.org/, accessed on 29 December 2025).

In determinations of the reaction rate between tRNA and AgNPs, the samples contained 1 μM tRNA in 20 mM Tris, containing 0.2 mM Mg^2+^, 5 μM ATP, in the absence or presence of 50 μM TCEP. To establish the effect of Mg^2+^ ions on the kinetics of AgNPs dissolution, a 20 mM Tris buffer, containing 5 μM ATP was used, with the spectra recorded every 3 min. Various MgCl_2_ concentrations between 0.5 and 3 mM were tested.

### 4.5. Fluorescence Spectroscopy

Fluorescence emission spectra of EMAP II were measured using a Cary Eclipse spectrofluorimeter (Varian Inc., Palo Alto, CA, USA) at 25 °C, using the excitation wavelength of 280 nm. The fluorescence was recorded in the range of 300 to 400 nm, with 5 nm or 10 nm slits for excitation and emission. Continuous stirring was used during titrations. Measurements for tRNA binding to the protein were performed at 35 °C. Titration fluorescence measurements were performed at 25 °C under constant stirring. Temperature dependence (melting) experiments were performed in the temperature range of 32–50 °C, with the use of a built-in Peltier unit.

#### A Note Regarding Temperature Dependence of Buffers and EMAP II pI

The temperature dependence of pH of Tris and Hepes buffers, used in melting experiments is −0.025 units/degree [44] and −0.014 units/degree [45], respectively. The temperature dependence of pI of EMAP II is expected to exhibit the analogous effect. By analogy with the histidine buffer, it can be estimated at −0.01 units/degree [46]. The pH of the working buffer at $T_{0}$ = 25 °C is 8.0 and the pI of EMAP II is 7.06, as estimated by the Protparam program [47]. The ΔT from $T_{0}$ to $T_{1/2}$ ∼45 °C is 20 degrees, and to the final temperature it is 25 degrees. The estimated values are presented in Table 1.

Based upon these values, one can conclude the pH gap between the pI and the buffer is sufficient to avoid affecting the protein stability by changing its surface protonation pattern.

### 4.6. NMR Spectroscopy

Heteronuclear NMR experiments were performed on Varian Inova 500 (^1^H frequency 500.606 MHz) NMR spectrometer operated at magnetic field 11.7 T, equipped with three channels, ^1^H/^13^C/^15^N triple resonance probe with inverse detection, and *z* gradient unit. The experimental data were acquired at 25 °C. The experimental sample contained 150 μM uniformly ^15^N-labeled EMAP II protein was prepared in 90%/10% H_2_O/D_2_O, 20 mM Tris buffer, 100 mM NaCl, pH 8.0. Referencing of the ^1^H, and ^15^N resonances were perform indirectly to external sodium 2,2-dimethyl-2-silapentane-5-sulfonate (DSS) using the 0.101329118 ratios for ^15^N [48]. The paramagnetic contact agent CrAcAc (Chromium(III) acetylacetonate) was added to the sample (10 μM) to identify the surface residues of the protein. All spectra were processed with the NMRPipe [49] and analyzed with the NMRFAM-SPARKY (version 1.470) [50] software. Sequence-specific backbone assignments of the ^1^H and ^15^N resonances were done on base previously published data [40] deposited in bmrb databank under accession code 18045.

## 5. Conclusions

Investigation into the molecular effects of AgNPs on protein conformation and function is necessary for a comprehensive assessment of their interactions. Considering the wide application of AgNPs in healthcare and daily use products, it is important to investigate their biosafety. The analysis of structure and activity of individual proteins in the presence of AgNPs is an important element of this goal. In our research, we established the possible influence of AgNPs on the properties of the EMAP II protein, focusing on its ability to bind tRNA, a prerequisite for protein biosynthesis and a crucial element of the cell cycle. We have also shown that upon interaction with silver ions derived from the dissolving nanoparticles, the protein EMAP II loses its native structure, which is confirmed by fluorescence analysis. This in turn may lead to disruption of reaction cascades during tumorgenesis and antitumor therapy, since EMAP II is a promising therapeutic antitumor protein [51,52,53].

## Figures and Tables

**Table 1:** Temperature dependence of buffer pH and EMAP II.

Temperature	Tris	HEPES	EMAP II pI
25	8.0	8.0	7.06
45	7.5	7.72	6.86
50	7.38	7.65	6.81

## Data Availability

Experimental data available from the authors under request.

## Supplementary Materials

The following supporting information can be downloaded at: https://www.mdpi.com/article/10.3390/ijms27020605/s1.

## Abbreviations

The following abbreviations are used in this manuscript:

EMAP II	Endothelial monocyte-activating polypeptide II
AgNPs	Silver Nanoparticles
NP	Nanoparticles
ARS	Aminoacyl-tRNA synthetase
MSC	Multi-tRNA synthetase complex
AIMP1	ARS-interacting multifunctional protein 1
TCEP	tris(2-carboxyethyl)phosphine
PDI	Polydispersity Index
ESI-MS	Electrospray ionization mass spectrometry
DLS	Dynamic light scattering
tRNA	Transfer RNA
pI	isoelectric point
NMR	Nuclear Magnetic Resonance
HSQC	Heteronuclear Single Quantum Correlation
CrAcAc	Chromium(III) acetylacetonate
