# Supplementary material for: Dissolving Silver Nanoparticles Modulate the Endothelial Monocyte-Activating Polypeptide II (EMAP II) by Partially Unfolding the Protein Leading to tRNA Binding Enhancement
Source: Int J Mol Sci. 2026 Jan 7;27(2):605. doi: 10.3390/ijms27020605 (PMC12840782; doi:10.3390/ijms27020605)
Supplement: Supplementary file 1 [file ijms-27-00605-s001.zip › ijms-4034863-supplementary.pdf]

Supplementary Materials: Dissolving Silver Nanoparticles Modulate the Endothelial Monocyte-Activating Polypeptide II (EMAP II) by Partially Unfolding the Protein Leading to tRNA Binding Enhancement

Lesia Kolomiiets*, Paulina Szczerba, Wojciech Bal and Igor Zhukov*

Characterization of EMAP II, tRNA and AgNP

The recombinant EMAP II protein was expressed in *E. coli* strain BL21(DE3)pLysE and purified chromatographically as described in the Materials and Methods section. Its homogeneity and correct molecular mass were confirmed by ESI-MS (Figure S1). The correct folding of the protein was confirmed by NMR (Figure S3). The size of nominally 20 nm citrate-coated silver nanoparticles (AgNP) were controlled with DLS, as presented in Figure S4. The obtained distribution of AgNP sizes ranged from 30 to 40 nm, with a mean diameter of 33.9 nm (Polydispersity Index PDI = 0.24). This value corresponds to the sum of metallic core and citrate coating dimensions, as demonstrated before. The zeta potential of citrate-coated AgNP was –36.9 mV, while for EMAP II alone it was –4.7 mV. The mixture of 1 μM EMAP with the AgNP stock diluted by adding 150 μL to the final 700 μL sample (ca. five-fold) was characterized by z = –7.8 mV to –9.6 mV, which indicates tight coating of AgNP with the protein. This was corroborated by the increased mean hydrodynamic diameter of AgNP particles in this sample, to 48.35 nm (PDI = 0.16). The average diameter of the EMAP II molecule by DLS was 8.2 nm (Figure S4C). Taking into account the total Ag concentration in the stock solution delivered by the supplier was 188.95 μM, the 150/700 dilution yields the total solution silver (Ag^0 + Ag^+) of 40 μM. Assuming the AgNP core size of 20 nm, one obtains the total concentration of AgNP of 165 pM, at ca. 250 000 Ag atoms per AgNP.

Table S1. Melting temperatures ($T_{1/2}$) derived from the sigmoidal fitting of frluorescence maximum position vs. temperature for 1 μM EMAP II in 20 mM Tris or 20 mM Hepes, containing 0.2 mM $MgCl_2$, 5 μM ATP, 50 μM TCEP, pH 8.0, in the absence and presence of 1 μM tRNA

Buffer	EMAP II	EMAP II + tRNA
Tris	45.3 ± 0.2	44.7 ± 0.2
Hepes	38.4 ± 0.4	41.7 ± 0.4

Table S2. Affinity constants for tRNA binding to EMAP II denatured by AgNPs, calculated for individual titrations presented in Figure S16

Panel in Figure S16	EMAP II (μM)	Log K_a	K_d (μM)
**with TCEP**			
**A**	1.00	6.24 ± 0.07	0.60 ± 0.10
**B**	1.85	6.30 ± 0.40	0.55 ± 0.40
**C**	1.63	5.86 ± 0.06	1.40 ± 0.20
**without TCEP**			
**D**	1.00	6.14 ± 0.08	0.70 ± 0.15
**E**	2.10	5.97 ± 0.08	1.10 ± 0.30
**F**	1.85	6.25 ± 0.25	0.60 ± 0.30
**G**	1.90	5.97 ± 0.15	1.10 ± 0.40
**H**	1.89	6.15 ± 0.15	0.70 ± 0.30
**I**	1.89	6.11 ± 0.13	0.80 ± 0.20

For all the data presented in Table, $\log K_a = 6.11 \pm 0.14$, and $K_d = 0.8 \pm 0.3 \mu M$.

```
        10         20         30         40         50         60
GHMSKPIDVS RLDLRIGCII TARKHPDADS LYVEEVDVGE IAPRTVVSGL VNHVPLEQMQ

        70         80         90        100        110        120
NRMVILLCNL KPAKMRGVLS QAMVMCASSP EKIEILAPPN GSVPGDRITF DAFPGEPDKE

       130        140        150        160
LNPKKKIWEQ IQPDLHTNDE CVATYKGVPF EVKGKGVCRA QTMSNSGIK
```

Number of amino acids: 169

Molecular weight: 18543.55

Theoretical pI: 7.06

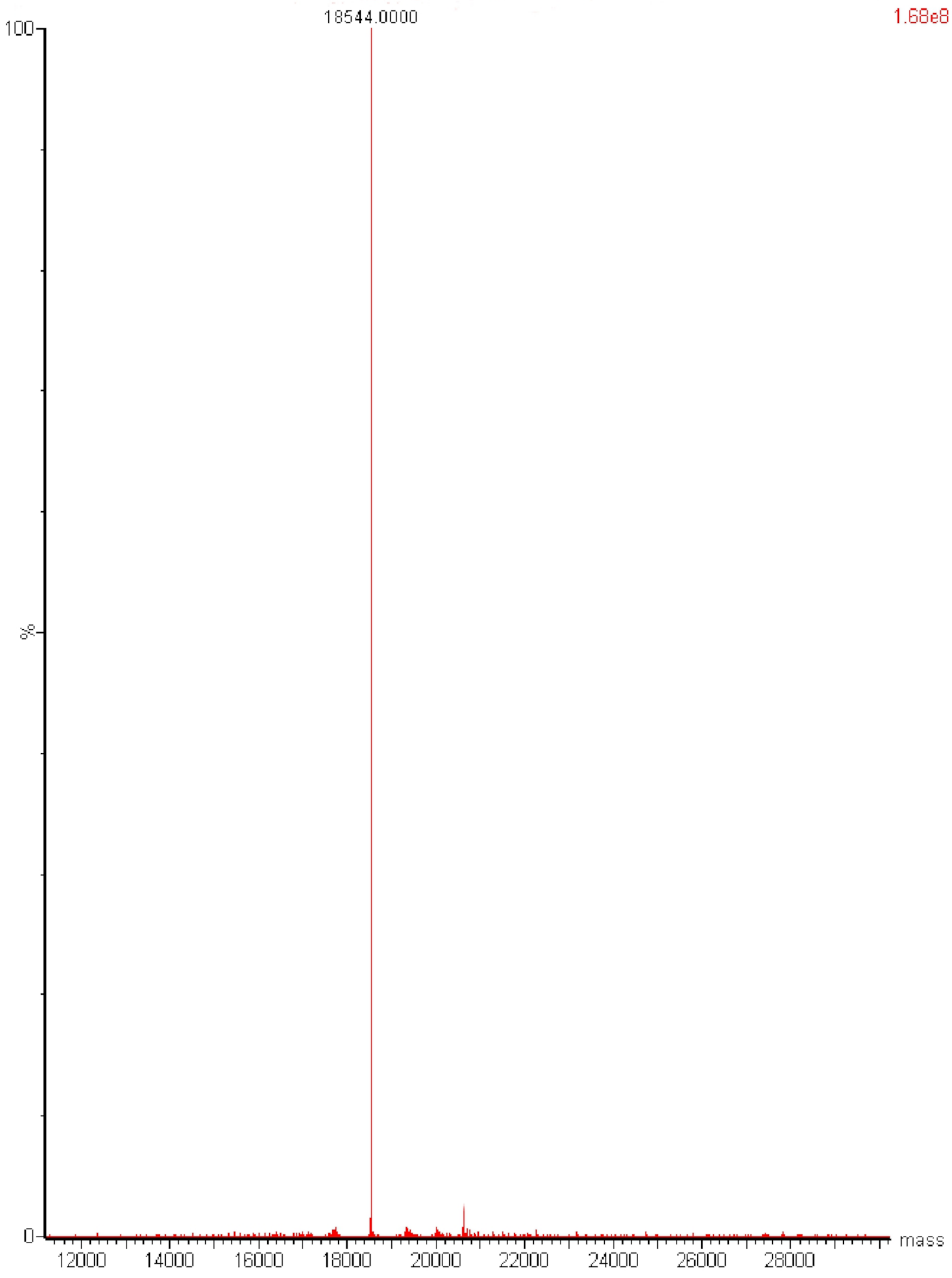

Figure S1. The monoisotopic EMAP II signal reconstructed from the ESI-MS spectrum recorded for EMAP II solution in 20 mM Tris, 150mM NaCl, 1mM DTT at pH 8.0 using an instrument resolution of 10000, capillary voltage of 2.5 kV, cone voltage of 40 V, and backing pressure of 1.95 mBar.

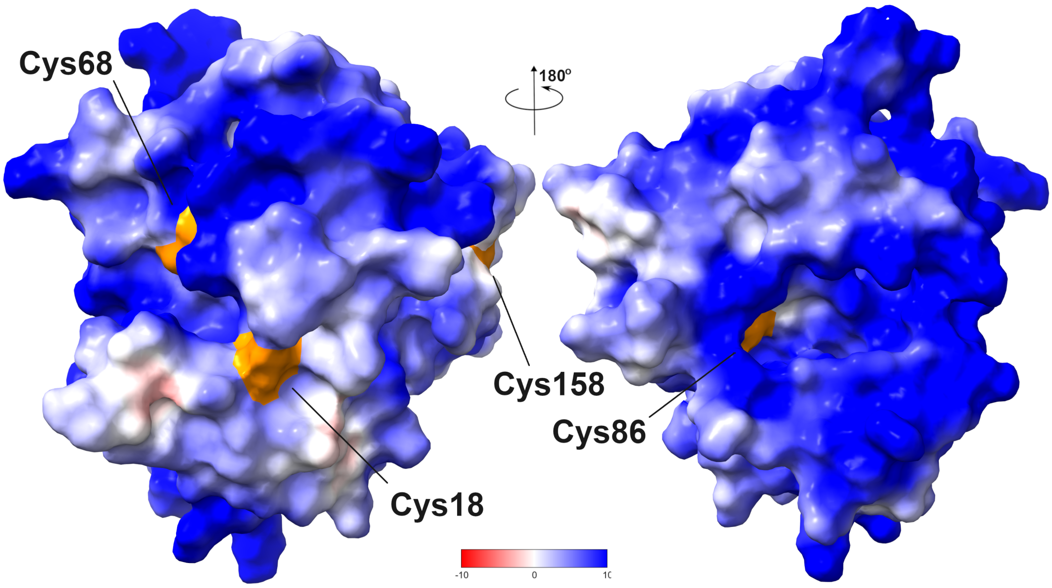

Figure S2. Surface presentation of the 3D structure of EMAP II protein in solution. Surface colored according coulombic potential calculated with ChimeraX program utilizing amber forcefield. The position of four out of five cysteines are shown in orange. The Cys141 buried inside hydrophobic core and do not has access to solvent.

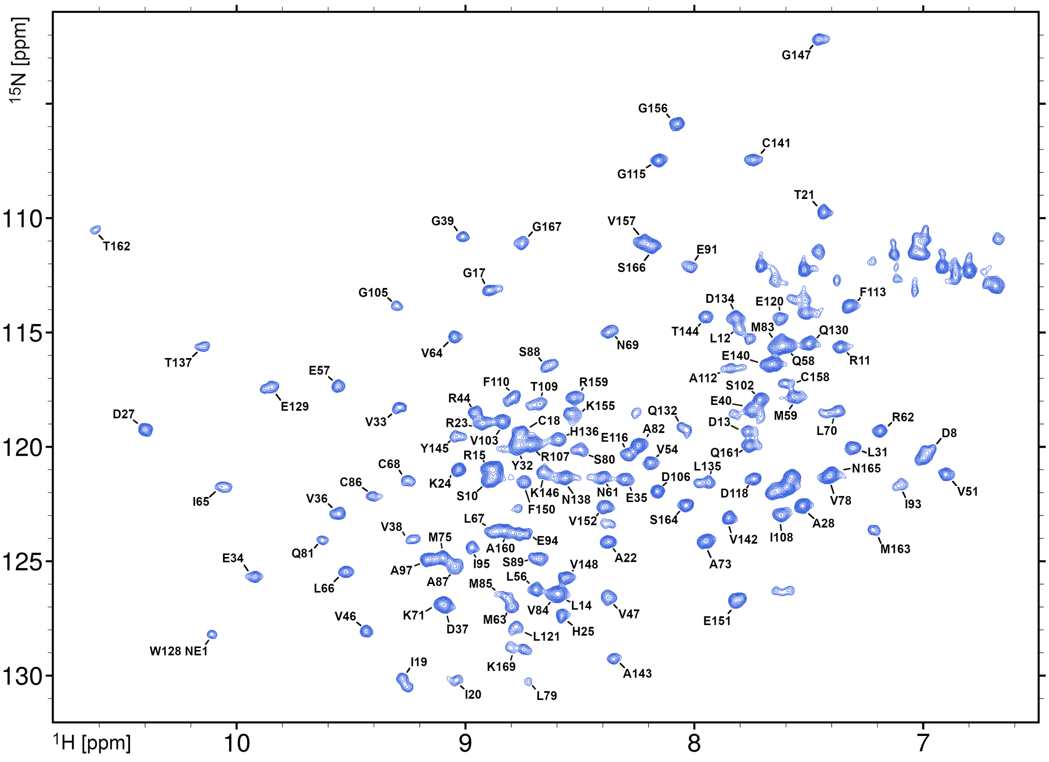

Figure S3. The ^{1}H-^{15}N HSQC spectrum collected for the uniformly ^{15}N-labeled EMAP II protein at 298 K utilizing Varian Inova 500 NMR spectrometer. The dispersion of signals from amide groups confirms the proper folding of the EMAP II protein in solution. The sequence-specific assignments achieved for the ^{1}H and ^{15}N backbone resonances are presented as one-letter code and sequence number.

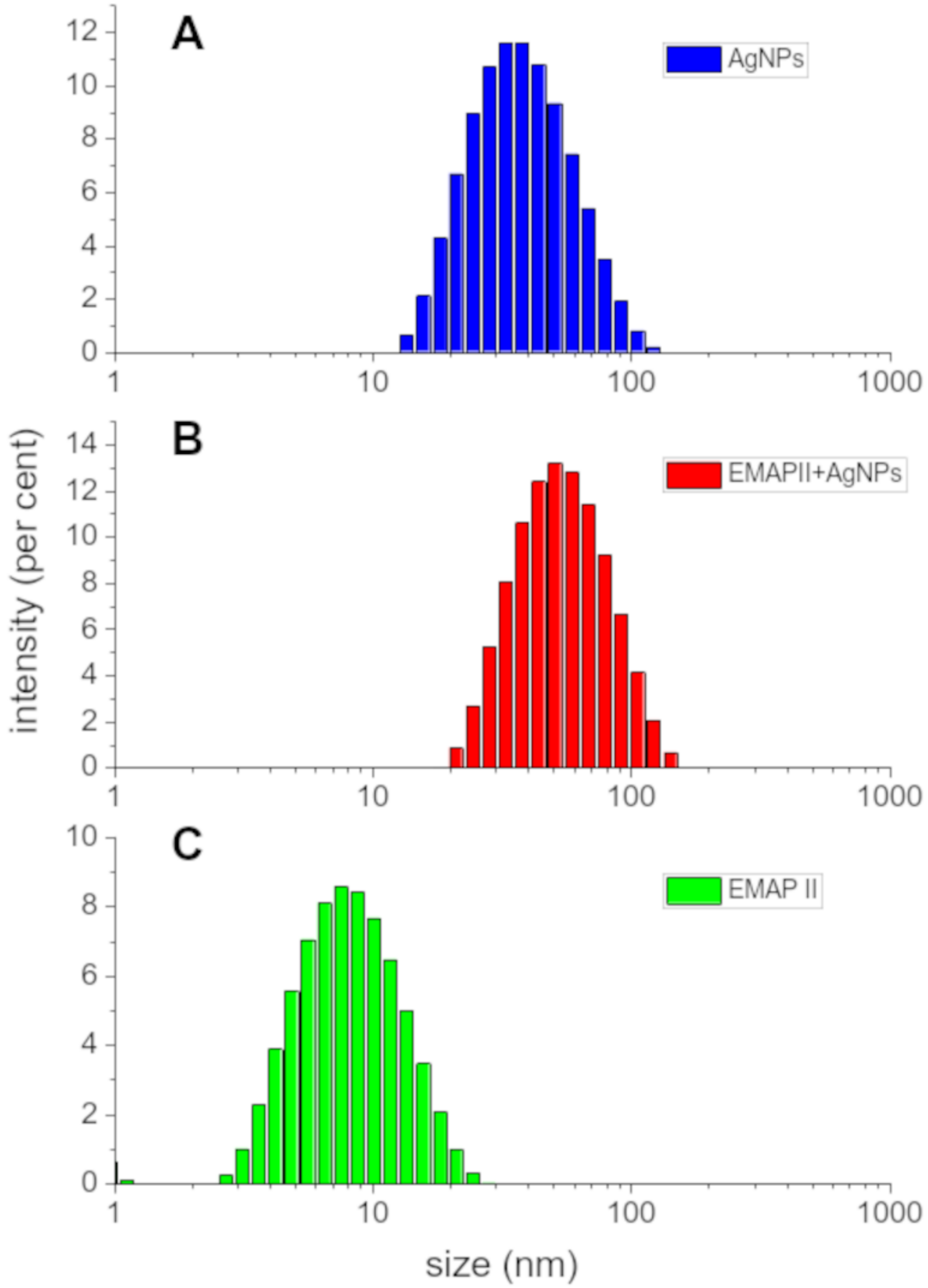

Figure S4. The particle size distributions measured by DLS for 165 pM AgNPs alone **(A)** and in the presence of 1 μM EMAP II in 20 mM Tris, pH 8.0 **(B)**; data for 1 μM EMAP II alone in the same conditions are given in **(C)**.

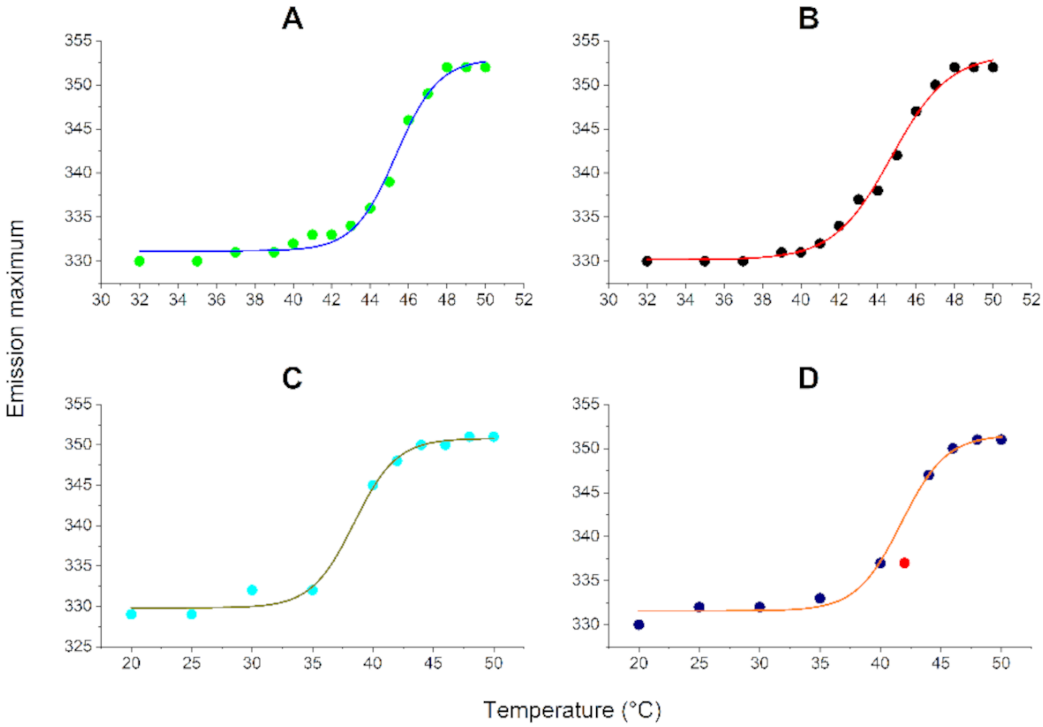

Figure S5. The melting curves derived from the fluorescence maximum for 1 μM EMAP II in 20 mM Tris **(A, B)** or Hepes **(C, D)**, 0.2 mM $MgCl_2$, 5 μM ATP, 50 μM TCEP, pH 8.0, in the absence **(A, C)** and presence **(B, D)** of 1 μM tRNA. Melting temperatures ($T_{1/2}$) obtained from sigmoidal fits (solid lines) are provided in Table S1.

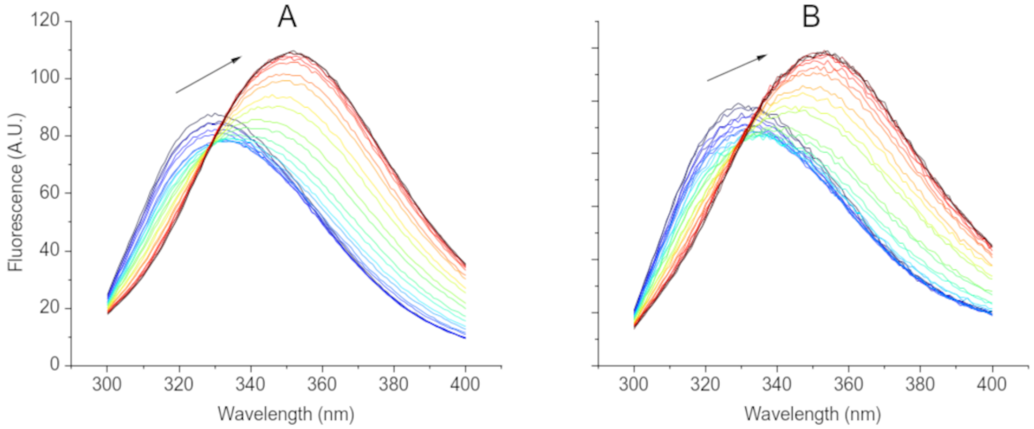

Figure S6. Temperature dependence of fluorescence spectra of 1 μM EMAP II in 20 mM Tris, 0.2 mM $MgCl_2$, 5 μM ATP, 50 μM TCEP, pH 8.0 in the absence **(A)** and presence **(B)** of 1 μM tRNA. The arrows indicate the direction of changes.

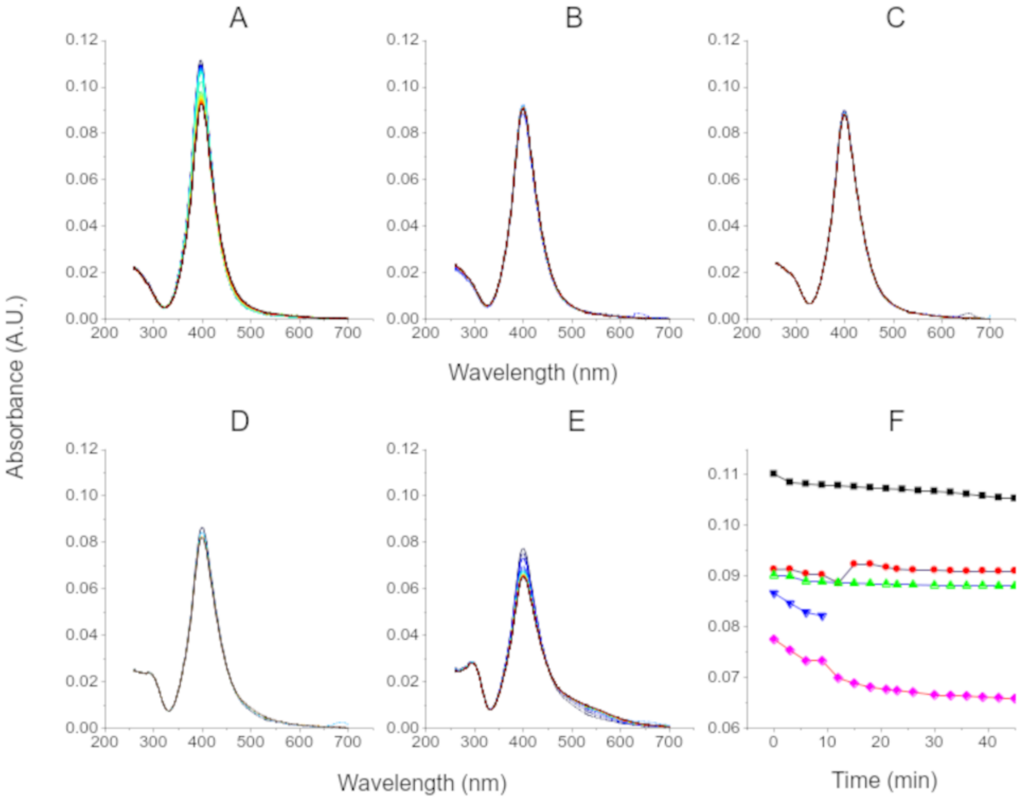

Figure S7. Decay of 82 pM AgNPs incubated in 20 mM Tris, pH 8 with various concentrations of Mg^{2+} ions from 0 to 3 mM, as indicated on graphs. **A)** 0 mM $MgCl_2$; **B)** 0.5 mM $MgCl_2$; **C)** 1 mM $MgCl_2$; **D)** 2 mM $MgCl_2$; **E)** 3 mM $MgCl_2$; **F)** Changes in the absorbance of AgNPs at a wavelength of 399 nm in the presence of different concentrations of $MgCl_2$. black: 0 mM, red: 0.5 mM, green: 1 mM, blue: 2 mM, magenta: 3 mM.

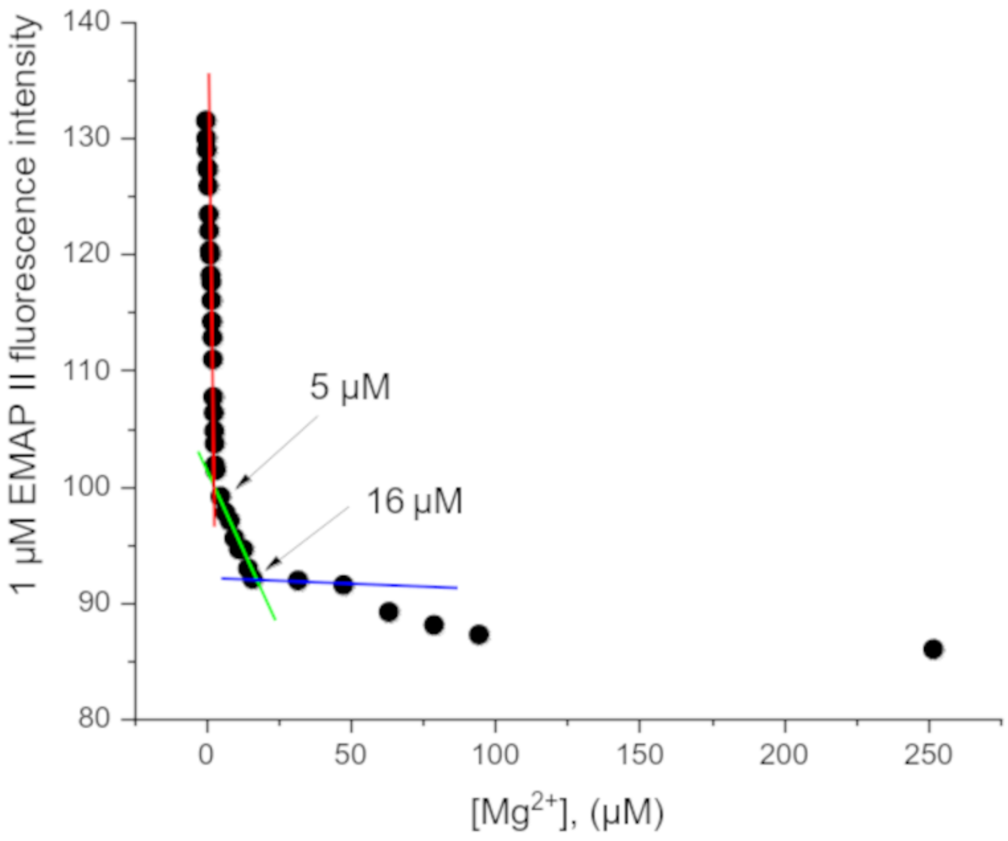

Figure S8. Partial quenching of fluorescence of 1 μM EMAP II in the presence of 1 μM tRNA in 20 mM Tris, 5 μM ATP, 50 μM TCEP, pH 8 upon the titration with Mg^{2+} ions. Key Mg^{2+} concentrations are given on the graph.

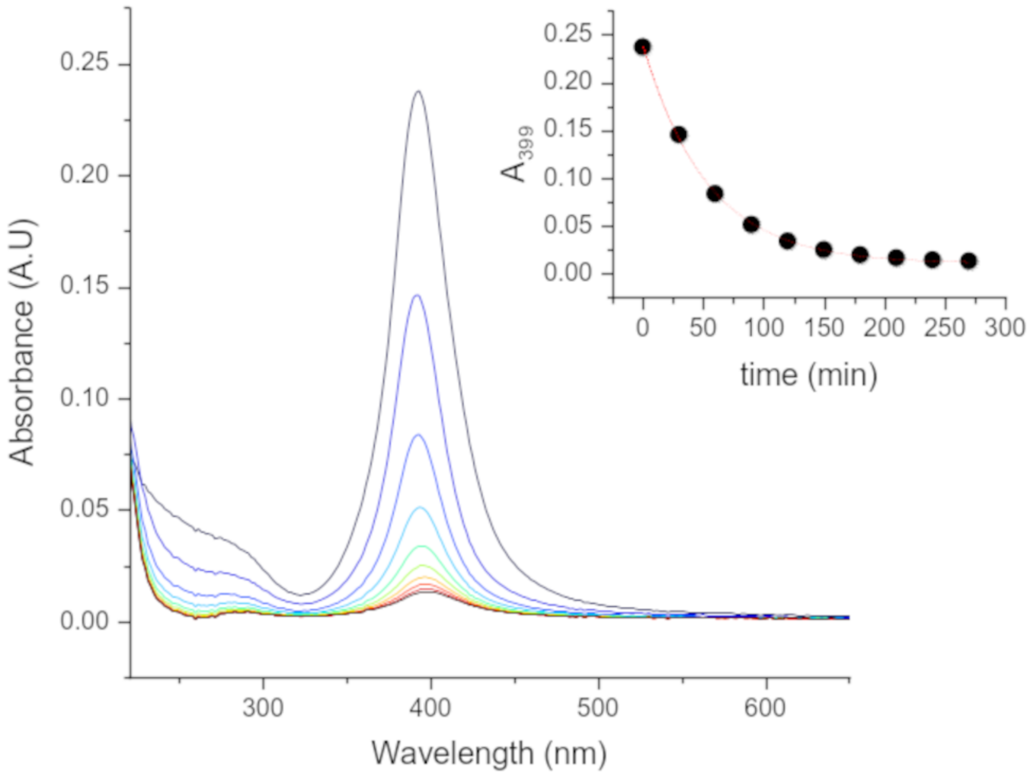

Figure S9. The effect of 50 μM TCEP on the stability of 165 pM AgNPs (40 μM Ag) in the optimized buffer: 20 mM Tris, 0.2 mM $MgCl_2$, 5 μM ATP, pH 8.

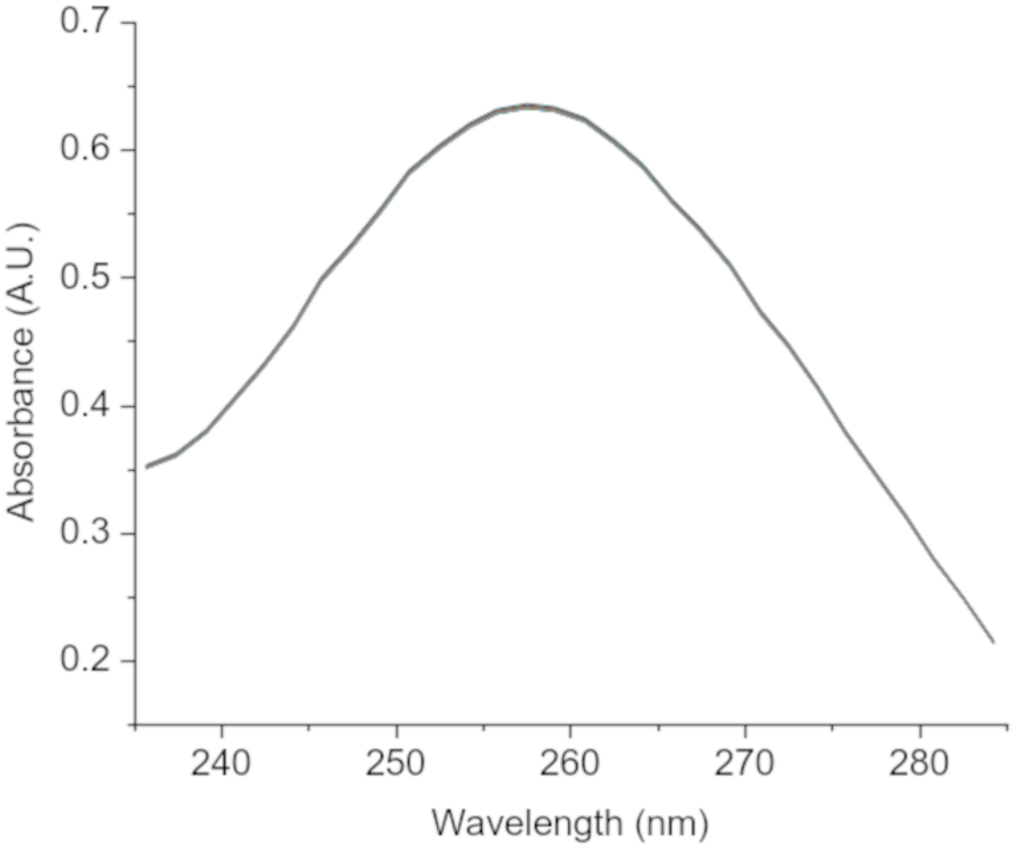

Figure S10. The stability of absorption band of 1 μM tRNA in the presence of 1 μM EMAP II in 20 mM Tris, 0.2 mM $MgCl_2$, 5 μM ATP, 50 μM TCEP, pH 8.0. The spectra were recorded every hour for 10 hours.

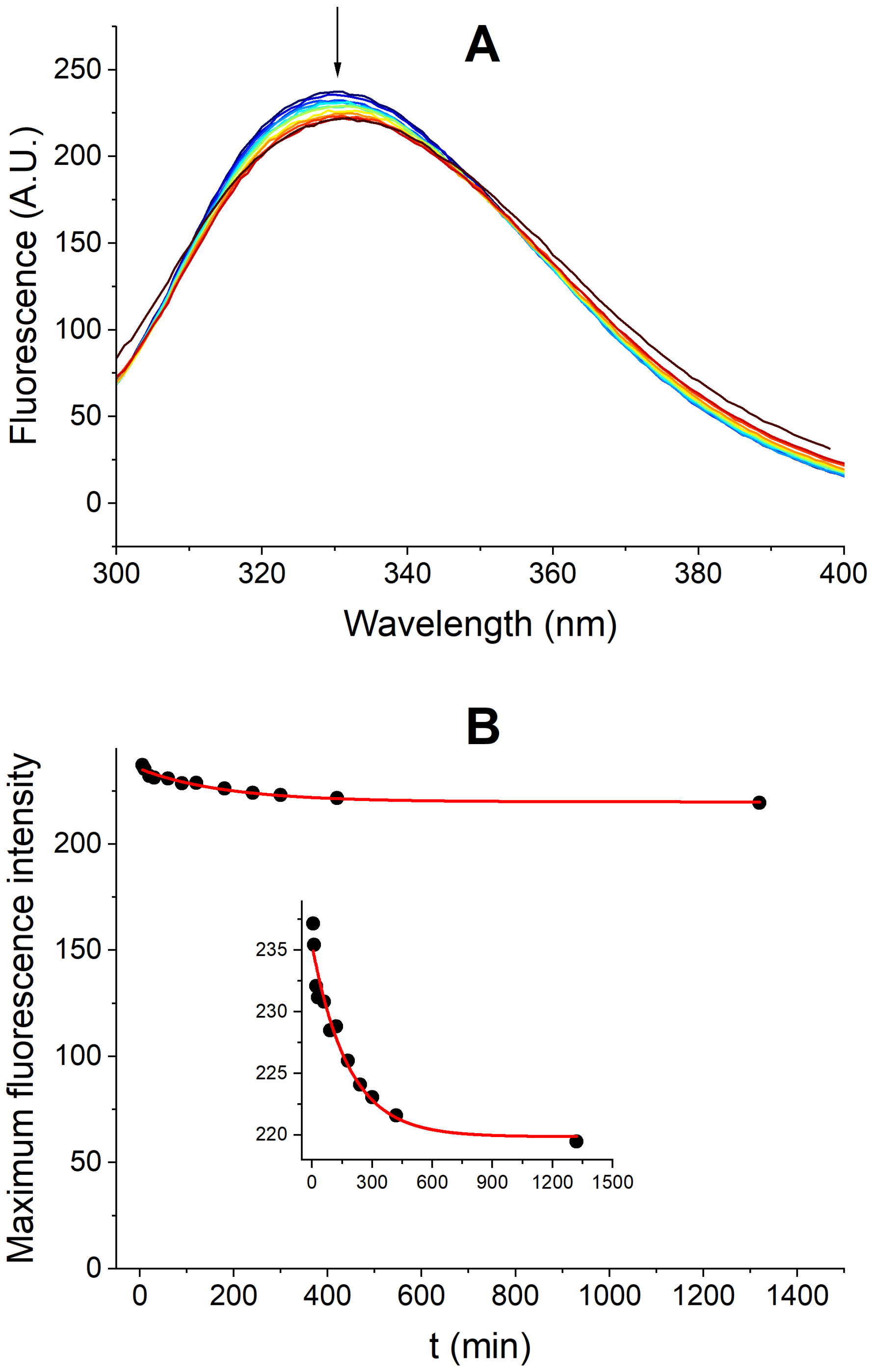

Figure S11. The evolution of fluorescence band of 1 μM EMAP II in 20 mM Tris, 0.2 mM $MgCl_2$, 5 μM ATP, pH 8.0 over 22 h incubation. **(A)** The spectra, arrow indicates the direction of changes. **(B)** Kinetic curve generated by plotting the fluorescence maximum vs. time. The red line represents the fit to the 1st order rate law.

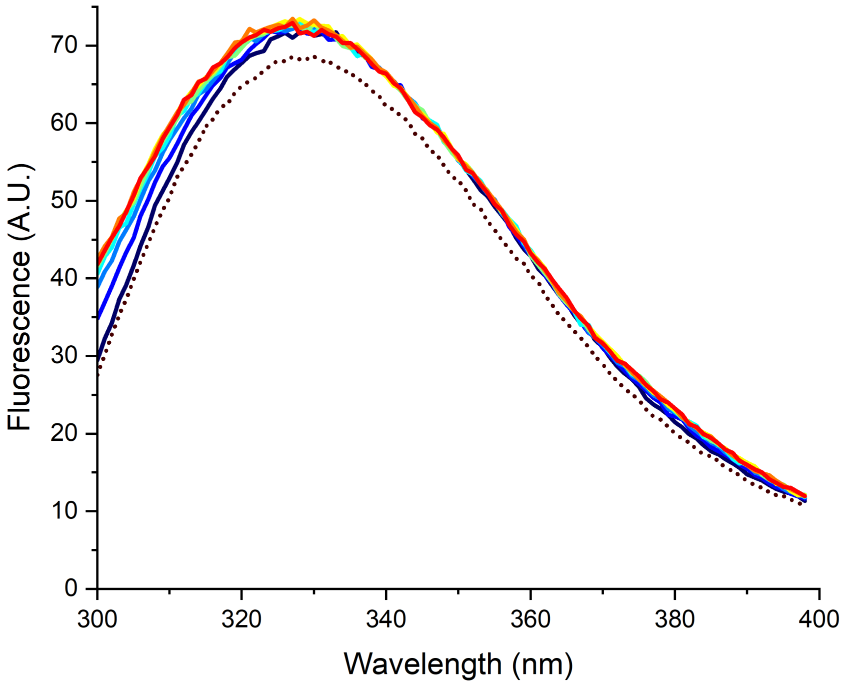

Figure S12. Time evolution of fluorescence band of 1 μM EMAP II in 20 mM Tris, 0.2 mM $MgCl_2$, 5 μµM ATP, pH 8.0, containing 1 μM tRNA. Solid lines: the spectra recorded every hour for seven hours. Dotted line: the spectrum recorded after 21 h of incubation.

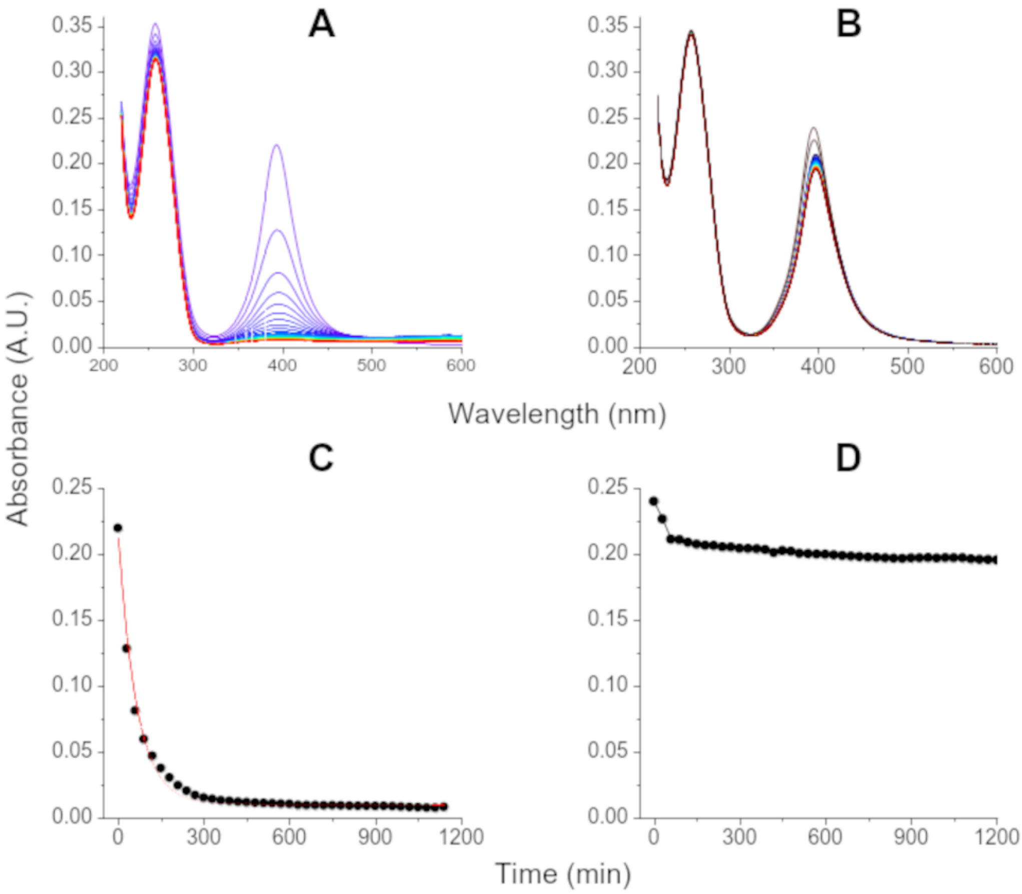

Figure S13. The kinetics of decay of 165 pM AgNPs in the presence of 1 μM tRNA in 20 mM Tris, pH 8.0, and the presence **(A)** or absence **(B)** of 50 μM TCEP. The kinetic plots at 399 nm for these reactions are presented in panels **(C)** and **(D)**, respectively. The 1st order kinetic fit in panel **(C)** is provided as red line.

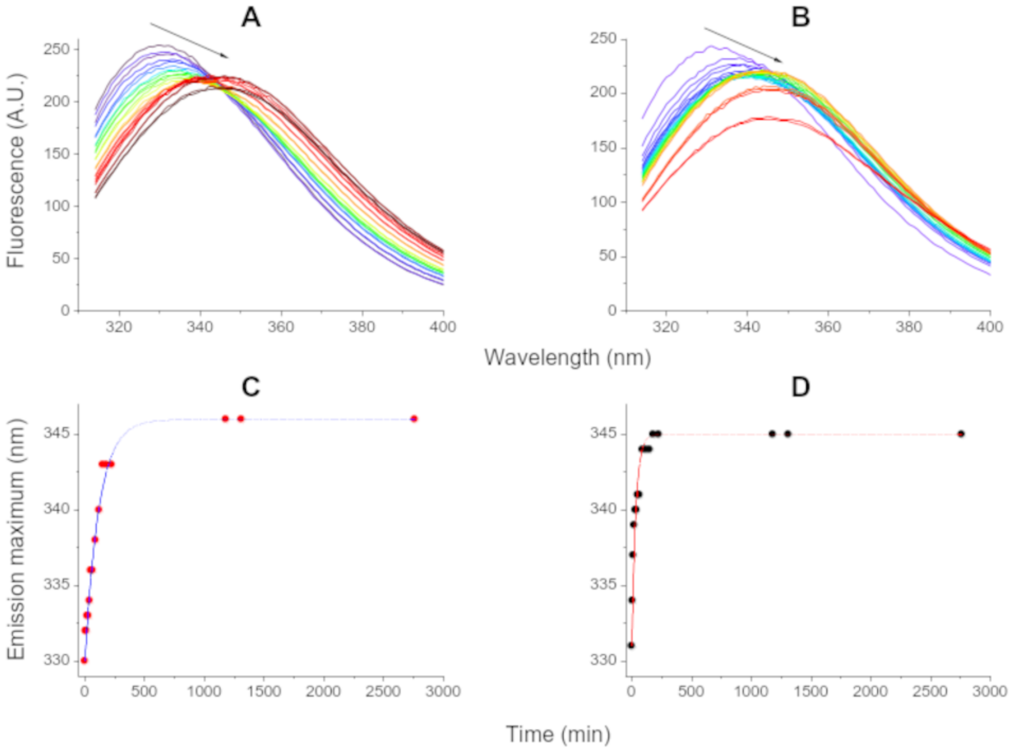

Figure S14. The kinetics of EMAP II interaction with Ag^{+} ions, delivered as 40 μM $AgNO_3$, in the optimized buffer, monitored by fluorescence spectroscopy over the period of 48 hours. Fluorescence spectra of 1 μM EMAP II were recorded periodically in 20 mM Tris, 0.2 mM $MgCl_2$, 5 μM ATP, pH 8.0, and the presence **(A)** or absence **(B)** of 50 μM TCEP. Arrows mark the general direction of change. The kinetic plots of emission maximum wavelengths for these reactions are presented in panels **(C)** and **(D)**, respectively. Lines mark the 1st order kinetic fits to these data.

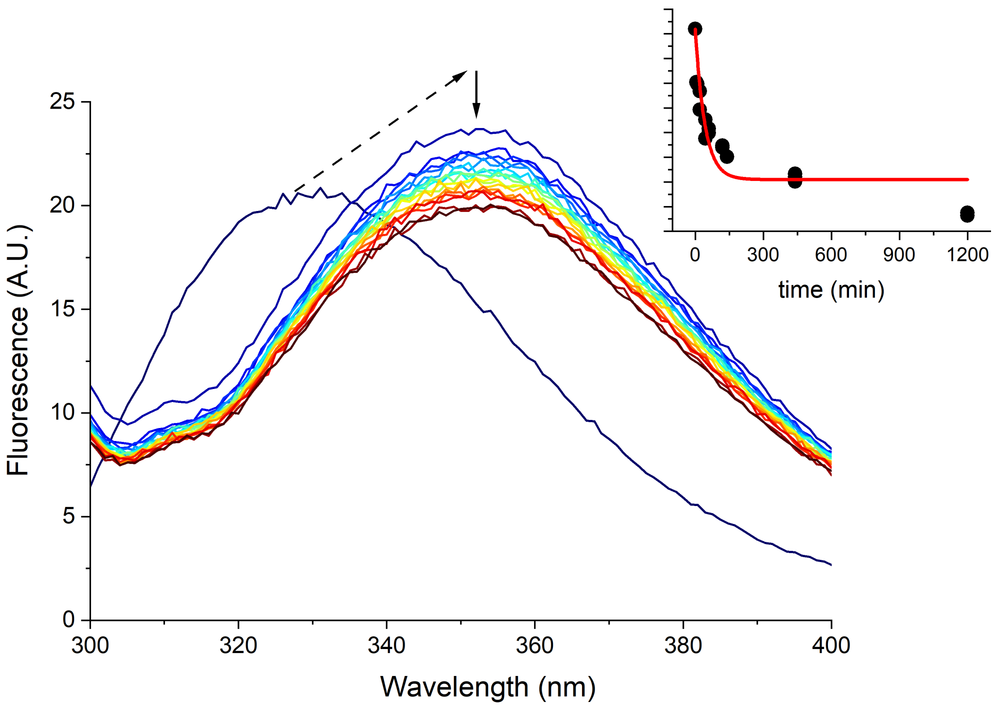

Figure S15. The kinetics of EMAP II/tRNA complex interaction with Ag^{+} ions, delivered as 40 μM $AgNO_3$, in the optimized buffer, monitored by fluorescence spectroscopy over the period of 20 hours. Fluorescence spectra of 1 μM EMAP II were recorded periodically in 20 mM Tris, 0.2 mM $MgCl_2$, 5 μM ATP, pH 8.0, and the absence of TCEP. Dashed arrow marks the immediate shift of the spectrum upon the $AgNO_3$ addition, while solid arrow indicates the direction of further spectral changes. The kinetic plot of emission intensity for the second phase of reaction is presented in the inset. The line marks the 1st order kinetic fit.

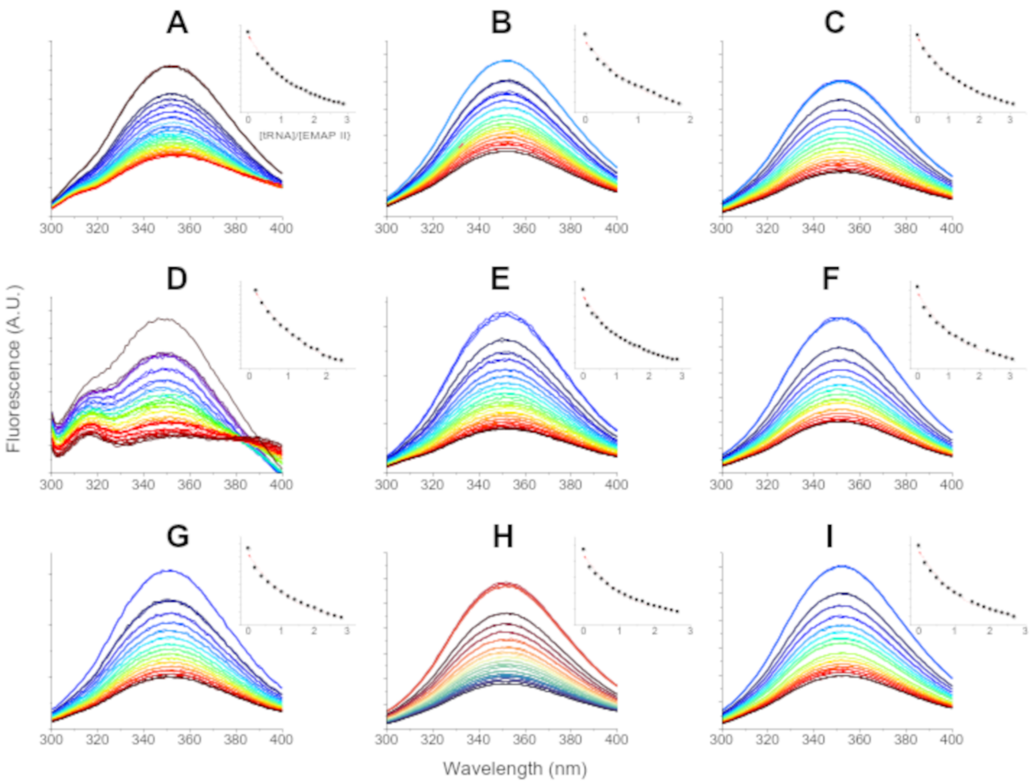

Figure S16. Fluorescence spectra of tRNA titrations of EMAP II after 24 hours incubation with 165 nM AgNPs in the optimized buffer (20 mM Tris, 0.2 mM $MgCl_2$, 5 μM ATP, pH 8.0), in the presence **(A-C)** and absence **(D-I)** of 50 μM TCEP. Protein concentrations in individual experiments are given in Table S1.

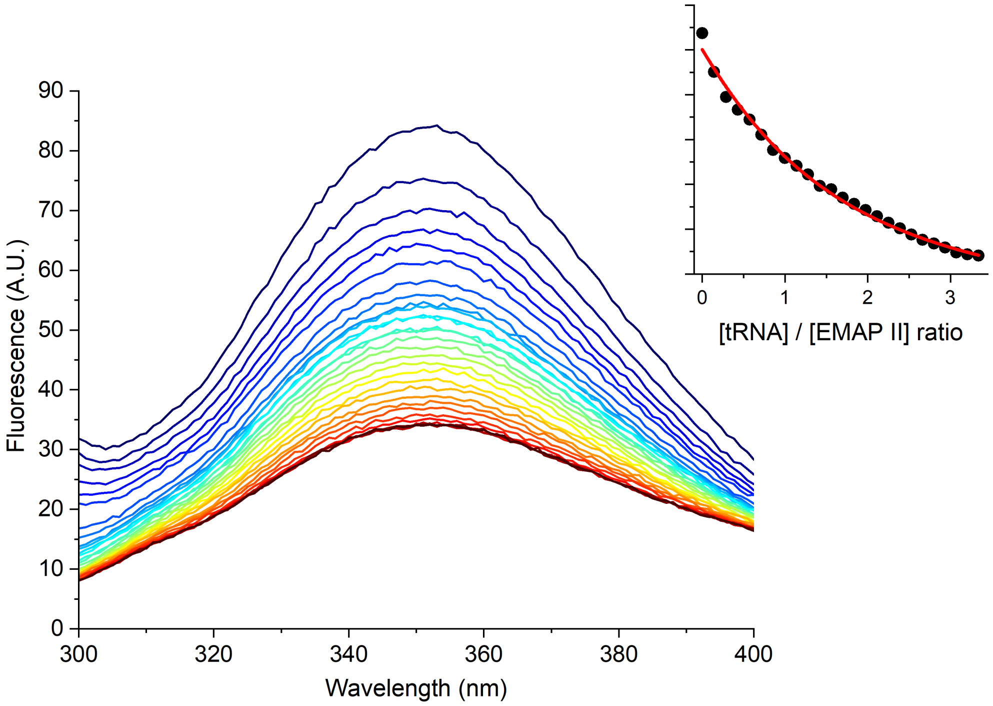

Figure S17. Fluorescence spectra and titration curve of tRNA titration of 1 μM EMAP II after 24 hours incubation with 40 μM $AgNO_3$ in the optimized buffer (20 mM Tris, 0.2 mM $MgCl_2$, 5 μM ATP, pH 8.0), in the absence of TCEP.
